# Inequality in the availability of residential air conditioning across 115 US metropolitan areas

**DOI:** 10.1093/pnasnexus/pgac210

**Published:** 2022-09-24

**Authors:** Yasmin Romitti, Ian Sue Wing, Keith R Spangler, Gregory A Wellenius

**Affiliations:** Department of Earth and Environment, Boston University, Boston, MA 02215, USA; Department of Earth and Environment, Boston University, Boston, MA 02215, USA; Department of Environmental Health, School of Public Health, Boston University, Boston, MA 02118, USA; Department of Environmental Health, School of Public Health, Boston University, Boston, MA 02118, USA

**Keywords:** residential air conditioning, climate change, social vulnerability, inequality

## Abstract

Continued climate change is increasing the frequency, severity, and duration of populations’ high temperature exposures. Indoor cooling is a key adaptation, especially in urban areas, where heat extremes are intensified—the urban heat island effect (UHI)—making residential air conditioning (AC) availability critical to protecting human health. In the United States, the differences in residential AC prevalence from one metropolitan area to another is well understood, but its intra-urban variation is poorly characterized, obscuring neighborhood-scale variability in populations’ heat vulnerability and adaptive capacity. We address this gap by constructing empirically derived probabilities of residential AC for 45,995 census tracts across 115 metropolitan areas. Within cities, AC is unequally distributed, with census tracts in the urban “core” exhibiting systematically lower prevalence than their suburban counterparts. Moreover, this disparity correlates strongly with multiple indicators of social vulnerability and summer daytime surface UHI intensity, highlighting the challenges that vulnerable urban populations face in adapting to climate-change driven heat stress amplification.

Significance Statement In the United States, residential AC is one of the most obvious and commonly available protective heat adaptation mechanisms, and potentially one of the most influential determinants of heat vulnerability. However, while inter-urban estimates of residential AC prevalence are broadly available for metropolitan areas, few studies have examined its *intra*-urban variation. This research provides census tract estimates of AC prevalence across a large sample of US cities with important implications for characterizing urban populations’ risk with regards to heat, and informing heat resilience policies and climate adaptation strategies, as well as future research on assessing the effect of heat adaptation on health outcomes.

## Introduction

The frequency and intensity of extreme high temperatures and heat spells, as well as the length and duration of season for extreme heat, are projected to increase further with continued climate change (1), posing an ongoing and increasing risk to the health of populations (2, 3). Extreme high temperature exposures are associated with increased morbidity and mortality (4–7), adverse mental health outcomes (8, 9), and decreased labor productivity (10). Temperature extremes are amplified in urban areas due to the influence of the built environment on radiative, thermal, moisture, and aerodynamic processes—the urban heat island (UHI) effect (11). Amongst personal cooling strategies (e.g. increased ventilation, use of electric fans, and evaporative coolers), residential air conditioning (AC) remains one of the most prevalent mechanisms of heat adaptation (12–14) and is crucial to reducing the risk of heat-related mortality (15, 16), increasing thermal comfort, and improving human capital accumulation and workplace productivity (17, 14, 18). The role played by access to AC in protecting human health against the adverse effects of extreme heat is therefore potentially large, but poorly characterized at the geographic scales at which such impacts manifest. Although estimates of AC prevalence are widely used in the development of heat vulnerability indices (12, 19, 20), evidence of its moderating effect on heat-related health outcomes has shown mixed results (21–24), largely due to the coarse spatial resolution of AC prevalence estimates that limit epidemiologic analyses (25, 26). Prior efforts to quantify residential AC have relied on survey data that vary in spatial scale and/or temporal resolution (27, 28, 24), focus on specific geographic areas (29–31), or lack information on AC adoption's fine-scale housing, socioeconomic and demographic correlates (32, 33). Moreover, broad regional or inter-urban comparisons of AC prevalence are not particularly actionable at the *local* level for which heat resilience planning and mitigation efforts are implemented. It is this need to comprehensively characterize *intra*-urban variation in residential AC availability, its origins, and consequences, that we address here.

In this paper, we use longitudinal survey data from the American Housing Survey (AHS) and the American Community Survey (ACS) to construct probabilities of household availability of AC at the census tract level in 115 core-based statistical areas (CBSAs, hereafter, “metropolitan areas”) that are in aggregate home to 67% of the total US population. We focus explicitly on AC prevalence in residential settings given that many adverse heat-related health outcomes are associated with indoor temperature exposures (34) and the home is where most people spend the majority of their time, though we acknowledge that heat exposures and AC usage also occur elsewhere (e.g. school or work). The resulting variability in the spatial distribution of residential AC within cities demonstrates a pattern of inequality in AC prevalence that is pervasive across metro areas: “core” high population density urban census tracts tend to have lower prevalence of AC compared to their lower-density suburban and outlying counterparts, irrespective of regional climate. We show here that AC prevalence is inversely associated not only with overall social vulnerability, but also with surface urban heat island (SUHI) intensity (SUHI is a type of UHI that refers to urban-rural differences in land surface temperatures. This is distinct from UHIs that are, for example, measured by differences in air temperature between the surface and roof-level, known as the canopy layer urban heat island, CUHI) (35): within a metropolitan area, the tracts with the least amount of AC tend to have both greater social vulnerability and SUHI intensity. While we are not able to causally link these attributes of heat vulnerability with adverse health outcomes within a given city, our results suggest further evidence that extreme heat risks are systematically unequally distributed within US cities.

## Results

Following the framework used by the Intergovernmental Panel on Climate Change (IPCC) (36), we refer to *risk* in the context of climate change impacts as embodying the dynamic intersection of *hazards* (a climate event or trend that impacts society or ecosystems—here, extreme heat), *exposures* (entities at risk—populations and human health), and *vulnerabilities* (characteristics that increase the propensity of a negative outcome conditional on experiencing a hazard—e.g. lack of AC, advanced age, and access to medical care). We evaluate census tract AC prevalence alongside several individual and aggregate markers of social vulnerability, as well as summer daytime SUHI intensity (which we use as an indicator of heat exposure amplification due to urbanization) to conceptualize the within-city spatial distribution of risks from heat.

### Census tract residential AC

We estimate AC prevalence as the probability of a household having either room or central AC, which we empirically project for 45,995 census tracts across US metro areas (details in the “Materials and Methods” section); suburban and rural tracts are not considered in our predictions. Urban prevalence of any residential AC (henceforth, “AC”) is generally high, with a median predicted probability of 0.97 and a range of 0.15 to 1.00 across census tracts and a population-weighted metropolitan area-level median predicted probability of 0.98 with a range of 0.28 to 1.00. Metropolitan-level estimates largely agree with the computed multiyear prevalence estimates of residential AC from the American Housing Survery (AHS, Supplementary Fig. S1), with an average absolute percentage deviation of 7.9% for metro area-level predictions. The largest differences between AHS and our predicted probabilities are observed in the Northeast, Northwest, Midwest, Colorado, and coastal California—cities that experience relatively cool climates with the fewest cooling degree days (CDDs)—the first and second quintiles of the distribution, and exhibit the most variability in residential AC within metropolitan areas. See Supplementary Fig. S2 for a map of CBSAs and their CDD quintile classification.

**Fig. 1.**
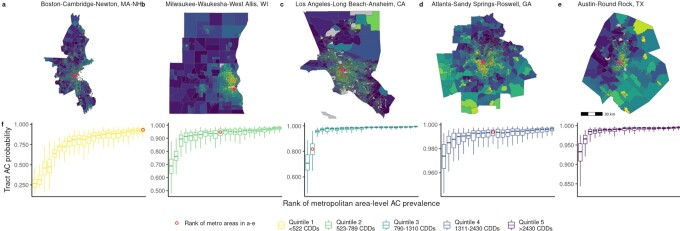
Intra-metropolitan area variation in percentile ranking of predicted probability of residential AC in five metro areas (a to e), each representing a quintile of regional climate (annual metropolitan area cooling degree days). Pink dots denote the metro area downtown or financial center. 5th percentile, median, and 95th percentile of metro area probabilities (f), grouped by CDD quintile.

Notwithstanding the generally high level of prevalence in cities across the country, AC is unequally distributed *within* cities. Rank ordering census tract probabilities of AC into percentiles within each metro area (see the “Materials and Methods” section) reveals a pattern of pervasive inequality in the spatial distribution of AC. Tracts in the urban core tend to have systematically lower AC prevalence compared to surrounding suburban tracts, while other outlying tracts have a tendency toward low to middle ranked probabilities of AC that depend on the metro area’s geographic, climate, and socioeconomic context. We illustrate this point by highlighting results for a representative metropolitan area from each CDD quintile, Fig. 1a to e (remaining metro areas are shown in Supplementary Fig. S3). This unequal distribution of AC is visible across metro areas in different CDD quintiles. Within-city variation of tract AC prevalence is largest for metro areas in the coolest climates and progressively declines with increasing CDDs, illustrated in Fig. 1f. The greatest relative variabilities in tract AC probabilities are found in cities in California and in Seattle, WA.

### Residential AC and social vulnerability

**Fig. 2.**
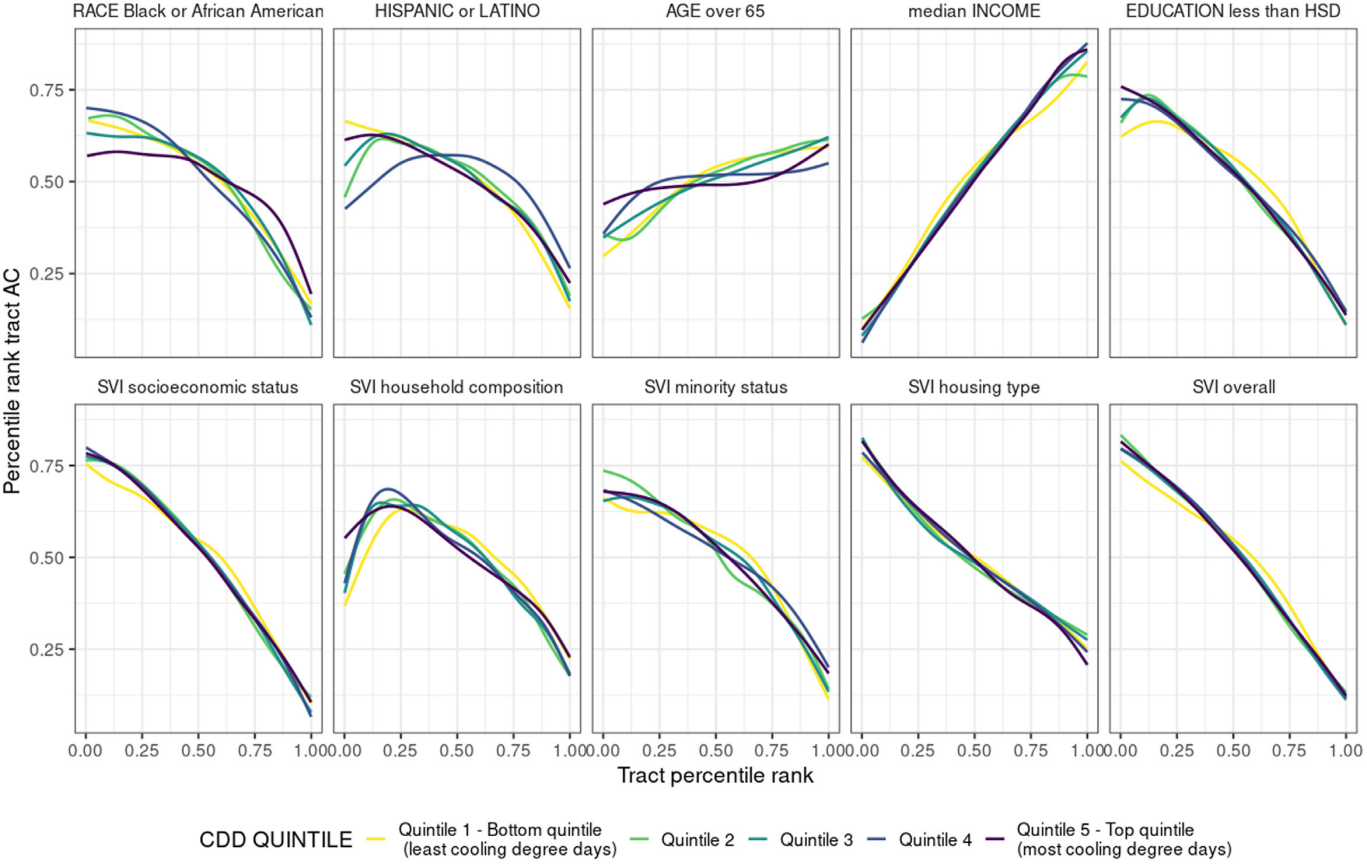
Percentile rankings of census tract AC and percentile ranking of select sociodemographic variables (top row) and SVI sub-indicator and overall scores (bottom row), grouped by CDD quintile of census tract metropolitan area.

Within each metropolitan area, our results illustrate that differences in the spatial distribution of residential AC prevalence are correlated with indicators of social vulnerability—race, ethnicity, age, income, educational attainment, and the composite social vulnerability index (SVI) (37). Across metro areas, the probability of AC is inversely correlated with the fraction of the population that identify as either Black or African American or Hispanic or Latino (Fig. 2). This pattern is somewhat weaker in warmer metro areas in the Southeast, Southwest, Texas, and inland California—quintile 5 for race and quintile 4 for ethnicity. Tracts with the lowest median income and those with high percentages of residents with less than high school education are the least likely to have AC. The same pattern emerges for census tract SVI values for the socioeconomic status summary theme: the median SVI is 0.86 for tracts below the 20th percentile of AC prevalence, whereas the median SVI is 0.20 for tracts above the 80th percentile. High percentages of elderly residents are associated with higher probabilities of having AC, but this association is the weakest of the correlates examined.

Across the four constituent SVI themes, and for overall SVI, high vulnerability is negatively correlated with tracts’ percentile ranking of AC, for cooler and warmer metro areas alike. Household composition and disability exhibits a non-monotonic relationship with AC prevalence. The latter is lower for tracts with SVI scores near 0 (severely vulnerable) in comparison to comparably less vulnerable tracts (0> SVI score >0.25), which can be traced to the partially offsetting impact of larger shares of elderly residents who are more likely to have AC. (Tracts ranked above the 80th percentile for share of elderly residents have a median AC percentile ranking of 0.61.)

### Residential AC and urban heat exposure amplification

**Fig. 3.**
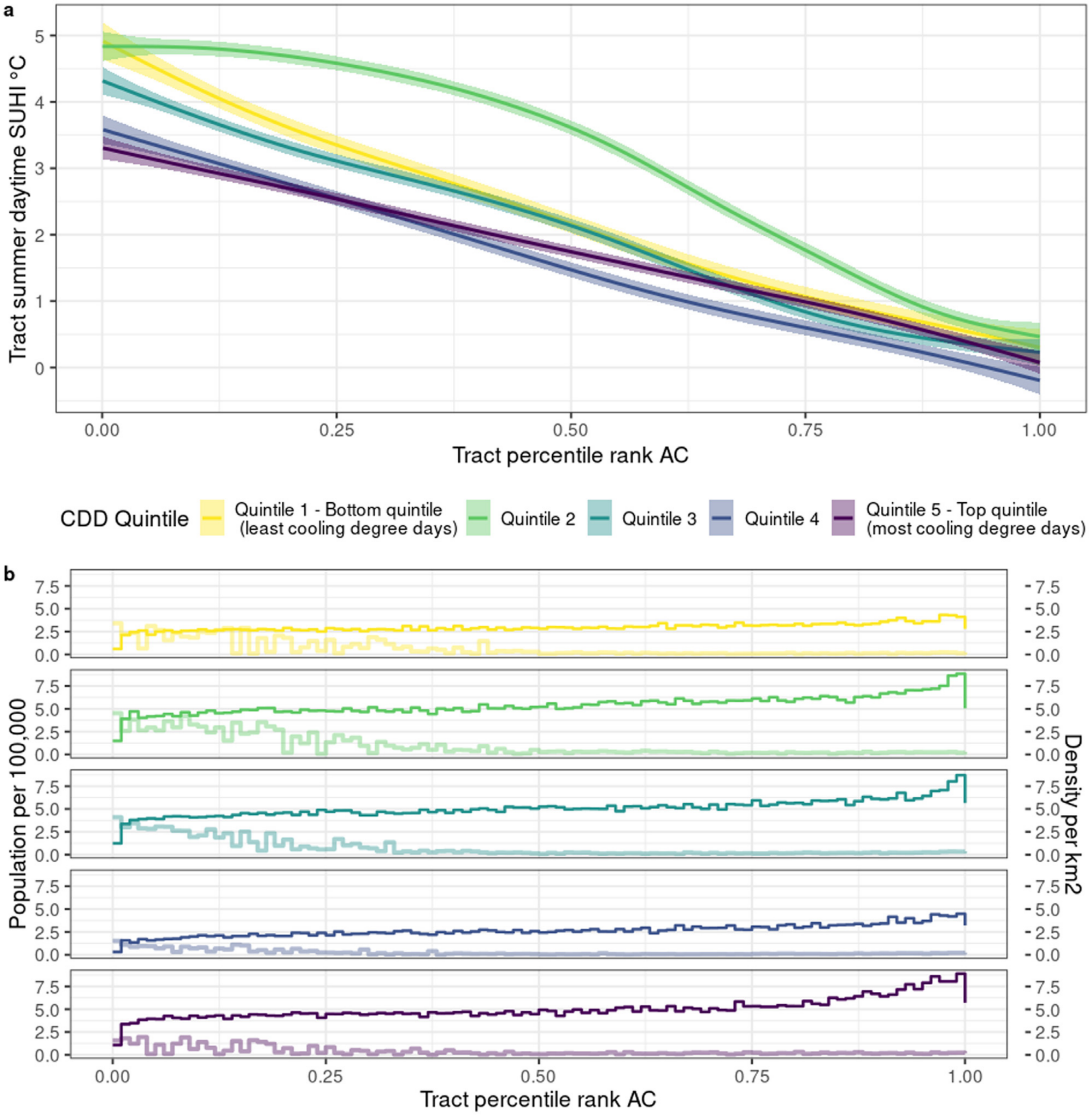
(a) Percentile ranking of census tract AC and summer daytime SUHI intensity (°C), grouped by CDD quintile of the metropolitan area. (b) Census tract population per 100,000 (dark color) and population density (light color) by CDD quintile and percentile ranking of tract AC.

Figure 3a contrasts summer daytime heat exposure amplification of census tracts, indicated by SUHI values, (38) with their within-city percentile ranking of AC prevalence. For our sample, median summer daytime SUHI is 2.3°C and ranges from −10.7°C to +10.6°C. Tracts facing the highest exposure to extreme heat amplification have systematically lower AC prevalence. Here as well, this trend is observed across metro areas in every quintile of CDDs. On average, tracts with low AC prevalence (<20th percentile) are 3.4°C hotter as compared to high (>80th percentile) prevalence tracts, and experience daytime SUHI intensities as high as 7°C over the year and 10.6°C in the summer. The highest summer daytime SUHI values (in excess of 8.7°C, > 99th percentile) occur in tracts of every CDD quintile but are predominately found in low AC prevalence tracts located in metro areas in the Northeast. High SUHI (>80th percentile), low AC prevalence tracts are the least populated but also the most dense (median population density 4.1 persons/km^2^) (Fig. 3b), which tend to be located in the urban core. Median population density for low AC tracts across all SUHI intensities ranges from 1.2 (quintile 4) to 3.9 (quintile 2) persons/km^2^.

Across US urban areas, the incidence of summer daytime SUHI exposure falls disproportionately on vulnerable communities (39). Patterns of disparities in residential AC mirror these spatial trends. Tracts that are identified as highly vulnerable under each of the four SVI summary themes or overall SVI (>80th percentile rank) and also exhibit low AC prevalence experience average summer daytime SUHI intensities of 4°C to 4.2°C, in stark contrast to −0.1°C to 0.7°C in low vulnerability (<20th percentile rank), high AC census tracts. Across CDD quintiles, these differences for overall SVI are largest for cities with the coolest climates, +5.1°C, and decline monotonically to +2.6°C in the top quintile.

### Intersection of residential AC, SUHI, and social vulnerability

**Fig. 4.**
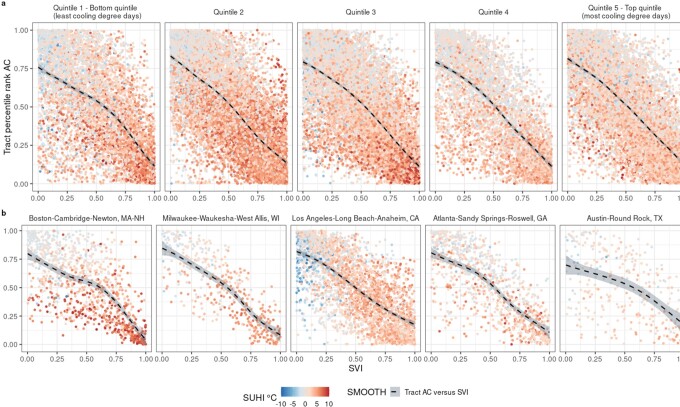
Intersection of AC, social vulnerability, and summer daytime SUHI intensity. (a) Smoothed trend of percentile rankings of census tract AC and census tract overall Social Vulnerability Index (SVI) score across metropolitan area CDD quintiles. Points are colored according to census tract SUHI intensities (°C), blue indicating the lowest intensities (surface cool island) and red indicating the highest intensities (surface heat island). (b) Census tract AC and SVI colored by SUHI for five illustrative metropolitan areas from each CDD quintile.

To provide a more comprehensive picture of overall risk associated with extreme heat, census tract AC prevalence, summer daytime SUHI, and social vulnerability are assessed together in Fig. 4 by CDD quintile (a) and illustrative metro area per CDD quintile (b). Tracts’ percentile rank of AC within a city, their overall social vulnerability, and heat amplification via SUHI demonstrate a negative linear relationship, irrespective of climate stratum or individual metro area. In particular, clusters of census tracts across metro areas in the third CDD quintile (Washington, D.C.; Baltimore, MD; St. Louis, MO; Louisville, KY; Lancaster, PA; and Philadelphia, PA) exhibit very high SUHI intensities (>7°C, 95th percentile), high social vulnerability, and low AC prevalence. Tracts in CDD quintile 1 show the largest dispersion of SUHI intensities across levels of AC and social vulnerability, a pattern that also arises to a lesser extent in CDD quintile 5. Additional clusters of tracts are highly vulnerable in terms of added temperature intensity and overall social vulnerability but less so in terms of adaptive capacity—between the 20th and 80th percentiles of AC—CDD quintile 2 (Bridgeport, CT; Hartford, CT; Chicago, IL; Detroit, MI; Milwaukee, WI; and New York, NY). Census tracts exhibiting high SVI and very high SUHI (>7°C) alongside high AC prevalence are only observed in New York, NY; San Francisco, CA; and San Jose, CA. While a clear inverse association between social vulnerability, SUHI intensity, and AC remains evident across our chosen set of example metro areas (Fig. 4b), the strength of this correlation across tracts is very heterogeneous. For example, numerous tracts throughout Boston, MA appear to have high SUHI across all levels of AC percentiles and SVI score, whereas there is a comparatively smaller range of SUHI intensity across tracts in Austin, TX.

Overall, while the combination of high social vulnerability, high SUHI (>80th percentile of summer daytime SUHI, 4.8°C), and low AC prevalence is characteristic of only a small fraction (4.7%) of urban census tracts, these high heat vulnerability areas occur in 70% of the metro areas in our sample and are, on average, among the most densely populated, with a total at-risk population of more than 7.8 million. Of the metro areas that include high heat vulnerability tracts, the fraction of the total population that is highly vulnerable to heat along all three dimensions ranges from 0.2% (Charlotte, NC, CDD quintile 4) to 11.9% (Boston, MA, CDD quintile 1), with a median of 3.2%. Cities where more than 10% of residents face high heat risk are predominantly located in the cooler climate of the Northeast.

Finally, because percentile rankings of AC prevalence do not reflect absolute AC probabilities, we additionally explore the intersection of high SUHI, high social vulnerability, and lower absolute probability of residential AC (<0.8) across census tracts, (Table 1). This grouping of vulnerability attributes is characteristic of census tracts in 17 of the 115 metropolitan areas in our sample and are primarily located in the Northeast, California, and Pacific Northwest (CDD quintiles 1 and 2). Of these, San Francisco has the largest vulnerable population along these components of heat vulnerability at more than 450,000, in contrast to only 4,155 in Akron, OH.

**Table 1.** Metropolitan areas with census tracts exhibiting low probability of AC (<0.8), high SUHI (>4.8°C), and high social vulnerability (overall SVI >0.8).

Metropolitan area	CDD quintile	Total population	Number of tracts	Affected population	Median AC probability	Median SUHI°C	Median SVI
San Francisco–Oakland–Hayward, CA	Quintile 1	4,588,807	93	458,600	0.39	6.45	0.90
Seattle–Tacoma–Bellevue, WA	Quintile 1	3,801,830	69	352,510	0.34	5.93	0.91
Los Angeles–Long Beach–Anaheim, CA	Quintile 3	12,751,313	67	289,805	0.76	5.55	0.91
Portland–Vancouver–Hillsboro, OR–WA	Quintile 1	2,437,447	43	230,650	0.75	5.53	0.94
San Jose-Sunnyvale-Santa Clara, CA	Quintile 2	1,952,215	30	161,047	0.73	5.23	0.91
Buffalo–Cheektowaga–Niagara Falls, NY	Quintile 1	1,116,840	35	111,111	0.66	7.10	0.90
Rochester, NY	Quintile 1	1,060,588	39	92,141	0.75	6.11	0.92
Providence–Warwick, RI–MA	Quintile 1	1,614,611	23	82,181	0.79	7.79	0.94
Worcester, MA–CT	Quintile 1	935,342	19	75,030	0.77	7.10	0.90
San Diego–Carlsbad, CA	Quintile 3	3,181,153	13	73,312	0.64	5.66	0.89
Syracuse, NY	Quintile 1	633,223	25	61,388	0.69	6.52	0.91
Albany–Schenectady–Troy, NY	Quintile 1	872,426	12	36,588	0.78	6.05	0.92
Utica–Rome, NY	Quintile 1	287,036	11	27,982	0.67	6.43	0.93
Eugene, OR	Quintile 1	364,408	5	24,504	0.60	5.23	0.96
Erie, PA	Quintile 1	270,003	7	16,308	0.72	7.07	0.91
New York–Newark–Jersey City, NY–NJ–PA	Quintile 2	18,304,055	1	5,050	0.69	5.15	0.92
Akron, OH	Quintile 2	697,627	2	4,155	0.79	5.30	0.81

Median AC probability, SUHI, and SVI denotes the median value of each characteristic of the tracts that meet the above defined criteria.

## Discussion

We demonstrate that across major US metro areas, census tracts in and around the urban core exhibit systematically lower percentile or relative rankings of AC prevalence compared to surrounding suburban and outlying census tracts, highlighting a fundamental inequality in intra-urban availability of residential AC that persists across diverse geographies and regional climates. Moreover, the pattern of inequality is strongly correlated with well-understood disparities in indicators of social vulnerability and amplification of summer heat exposure, with the potential for differential AC prevalence to exacerbate the risk of adverse heat-related health outcomes among vulnerable urban populations. Separately, while we do not estimate AC probabilities for suburban and rural census tracts, the mixed pattern of relatively high to mid- and low-ranking AC prevalence found in many suburban tracts across metro areas highlights the importance of characterizing nonmetro AC prevalence as an area of future study.

AC is currently one of the most widely available technologies to adapt to extreme heat by increasing thermal comfort and has been shown to be an influential factor in reducing heat-related mortality and improvements in workplace productivity (16, 14). It should be noted, however, that reliance on AC has simultaneously been shown to have important consequences for future electricity demand (40), the stability of the electric grid (41) (especially during heat waves), and anthropogenic heat fluxes (42). While alternative individual-level strategies for cooling such as increased ventilation and the use of electric fans have lower greenhouse gas emissions than AC, they may not be sufficient to offset physiological heat strain at high temperature extremes and are less effective amongst vulnerable groups such as older individuals (43, 14). Moreover, our focus on the *inequality* of residential AC prevalence highlights its significance as a determinant of disparities in heat vulnerability. Nevertheless, it is important that increased access to AC be energy efficient and increasingly powered by renewable energy sources alongside proliferation of other heat adaptation technologies (e.g. reflective coating, quality housing insulation), as well as passive and nature-based cooling approaches (e.g. shading infrastructure, greenspace).

Disparities in residential AC prevalence differ across geographies (12, 24) with underlying variations in the climatic, socioeconomic, and infrastructural determinants of adoption (44, 33, 45). US metro counties with high AC prevalence are estimated to have the lowest levels of heat vulnerability (12), which we corroborate. Across cities, there is a strong latitudinal gradient of prevalence of central AC, higher across the southern United States (24). Differences among metro areas in the prevalence of central and room AC are in large part predicted by housing characteristics (value, building age, and home ownership) as well as climate (32). Our study builds upon the work of Gronlund and Berrocal (32) by additionally considering the impact of demographic characteristics on the within-city variation in residential AC. A key challenge is that demographic data are not typically available at the parcel level. Thus, while it is possible to construct property-level predictions of the probability of AC ownership by coupling empirical models with assessor records of the structural characteristics of individual houses (32), we expand upon that approach to construct estimates that are spatially aggregated to the census tract and reflect a more complete set of influences. The advantage of the latter is that it allows us to undertake a comprehensive US-wide assessment of the implications of social and behavioral forces on vulnerability to extreme heat.

Compared to Sera et al. (24), our estimates of metro area AC prevalence are considerably higher, with a mean absolute percentage deviation of 45.2%, concentrated in CDD quintiles 1 and 2 (Supplementary Fig. S5). This phenomenon arises from several differences in our methodologies: predicting the distribution of central AC (24) rather than any AC, the use of different microdata sources in different regions, and a focus on an earlier period throughout which AC adoption was lower [additional details in Supplementary Information (SI)]. Results from Gronlund and Berrocal (32) highlight the potential to perform a US-wide cross-city comparison, but this opportunity has so far only been followed up on to a limited extent (41).

Separately, Gronlund and Berrocal (32) establish that AC prevalence varies within cities [see also Ito et al. (46)]. This literature heretofore has tended to focus on the effect of local and regional climatic, societal, and built-environment attributes on AC access (29, 28, 47, 32, 31) and its implications (48, 30) in a limited number of discrete locations. Compared to our findings, which include the additional effect of demographic correlates, application of Gronlund and Berrocal’s (32) structural-characteristics-only model to Detroit assessor data suggests a cross-tract median raw probability of any AC of 0.75, which is 10  percentage points lower than our estimate (Supplementary Fig.S6). We additionally apply their central AC model coefficients to our sample of cities to generate census tract probabilities with which to compare our estimates. Across tracts in the highest CDD quintiles, agreement between the two probabilities is high, with a mean absolute percentage difference of 2% and 2.3% for quintile 4 and quintile 5, respectively. Differences between estimates of AC prevalence increase sequentially across the remaining CDD quintiles, with the largest divergence in tract predictions of central AC compared to our estimates of any AC occuring in cities with the coolest climates (quintile 1) (details in the “Supplementary Material”).

While limited availability of replication data precludes additional head-to-head comparisons, our patterns of predicted probability of AC ownership are broadly consistent with prevalence documented in Fraser et al. (29), Guirguis et al. (28), Gamarro et al. (47), and Ahn et al. (31). For example, predicted probabilities for census tracts in Maricopa County (Phoenix metro area) are higher than those for Los Angeles County (LA metro area) mirroring the pattern described in Fraser et al. (29), and percentile rankings of tract prevalence in San Diego county suggest lower rates of AC along the coast, as in Guirguis et al. (28). In Jacksonville, Florida, Ahn et al. (31) find markedly lower AC ownership in the urban core, a result that we demonstrate persists throughout cities. (Detailed discussion in the Supplementary Material). Our contribution is to build on these efforts to construct comprehensive, consistent estimates of fine-scale AC prevalence for a broad range of climatically and socioeconomically diverse cities across the US.

A key implication of our results is that unequal AC prevalence compounds existing disparities in urban populations’ social vulnerability and temperature exposure. Together, social vulnerability and SUHI increase the risk of adverse heat-related health outcomes (49). Social vulnerability, as indicated by high SVI rankings, is both spatially clustered and correlated with heat-related morbidity (50, 51). Inner cities tend to experience higher surface temperatures (Supplementary Fig. S4), are more exposed to heat extremes, and their downtown cores are home to disproportionately larger shares of vulnerable populations (12). Surface temperatures are statistically higher in areas of poverty, ethnic minorities, lower education, and elderly residents (52) and Black, Hispanic or Latinx, and low-income populations disproportionately experience amplification of summer temperatures across both US and global urbanized areas (39, 53). Others have found that urban heat island intensity is disproportionately stronger in neighborhoods with more people of color and lower household incomes (54). Other spatial analyses have linked high surface temperature exposures across US urban areas to inequitable historical housing policy practices, i.e. “redlined,” neighborhoods that are overrepresented by poor and minority residents (55, 56).

Our results add to, and reinforce, this body of evidence, confirming the inverse relationship between minority—or low socioeconomic—status and residential AC prevalence (21, 27, 33); emphasizing that these vulnerable urban populations are systematically more likely to experience high temperatures but have systematically less capacity to adapt to this increasing heat than their more advantaged suburban counterparts. The latter will ultimately determine how the health consequences of heat exposure are distributed among the population. The present findings take the first step of elucidating the precursor distribution of adaptive capacity: elaborating the potentially unequal consequent moderating effects is well beyond the scope of our inquiry but is an active area of our research in progress, and relatedly, exploring city-level characteristics (particularly policies or practices) that are associated with lower inequalities in AC prevalence is of interest for future exploration.

It is important to recognize the limitations of our study. First, the multilevel nature of our empirical specification is slightly less flexible than the two-level structure employed by Gronlund and Berrocal (32), who capture the variation associated with regional climate using a continuous measure of CDDs that are also interacted with housing characteristics. We purposely opt for a more parsimonious specification in order to introduce additional housing and sociodemographic correlates that are known to drive AC adoption without creating potential pervasive multicollinearity. Separately, we would prefer to implement a multinomial model stratified by housing type (single or multifamily) in order to elucidate the joint probability of a household owning either central or room AC; however, sample size limitations for a number of metropolitan areas that result in a computationally singular system preclude the use of this approach.

Second, while housing characteristics are significant determinants of residential AC adoption (44, 32), our empirical model includes a limited subset of housing attributes that potentially predict household AC. In addition to housing type (single or multifamily), tenure (own or rent), and building age, detailed building properties, such as size (square footage of living space), insulation, and building quality have been shown to correlate with AC adoption (57, 45). Other physical attributes such as construction material, solar orientation, roof and wall albedo, and window-to-wall ratio modify indoor heat exposure and vulnerability (58), potentially indicative of the likelihood of having AC. The absence of comprehensive high-resolution data on such detailed housing information in the United States has restricted the inclusion of such variables in efforts to quantify intra-urban residential AC. While the AHS captures some information relating to building structural features (e.g. bedrooms, bathrooms, square footage, stories) and housing issues that reflect building quality (e.g. mold, leaks, heating problems), most of these variables are not captured by the ACS. Our subset of housing covariates therefore only reflects overarching building features such as type, tenure, and number of bedrooms (see the “Materials and Methods” section).

In addition, given the strategy of matching our empirical model’s covariates to the limited number of ACS tract-level building attributes, introducing additional explanatory variables into the empirical model might improve its precision, but cannot relax the binding constraint on the range of factors that can be brought to bear to project AC prevalence. In particular, the variables that AHS microdata have in common with other data sources such as ACS datasets also restrict our ability to investigate the potential impacts of a broader slate of relevant community, infrastructural, or institutional variables. Examples include proximity to greenspace (59), access to cooling centers (29), or improved housing quality (60), all of which indicate alternative means to keep cool that can potentially substitute for in-home AC use. A deeper caveat is that we estimate the probability of residential AC but cannot directly observe utilization conditional on ownership, which is the true measure of cooling access. Even if socially vulnerable households do have an air conditioner, their high energy costs and risk of energy poverty may render them unable to afford to operate it to the degree necessary to protect health (61–64). This issue is potentially important in the hot climates of the South and Southwest, where AC prevalence is quite high. For this reason, our transformation of AC probabilities into within-city percentile rankings may not fully capture urban populations capacity to adapt to high temperatures. Investigation of households’ ability to pay for cooling through the estimation of additional electricity consumption and its cost burden is another avenue of high interest for future work.

Third, we use a publicly available dataset of census tract-level remotely sensed SUHI as an indicator for urban heat exposure amplification, which does not necessarily reflect experienced indoor or outdoor human thermal comfort (38). SUHI intensity is based on land surface temperatures and, as such, is an imperfect metric of exposure [a detailed examination of SUHI in the context of urban heat is available elsewhere (65, 66)]. Other metrics, such as apparent temperature (ambient temperature, humidity) (67), wet-bulb globe temperature (ambient temperature, humidity, solar radiation, wind speed) (68), or ecostress (land surface temperature, evapotranspiration) (69) may more accurately capture absolute urban heat stress. However, our goal was to characterize *relative* spatial heterogeneity in AC prevalence and heat stress within each particular metropolitan area—in this context SUHI provides a useful metric for showing which parts of the city are relatively hotter than the rest of the city on summer-time days, and hence is an appropriate proxy for identifying places that may have greater AC needs relative to the city as a whole, net of other factors, irrespective of the absolute ambient temperature.

Finally, we acknowledge the concern that our focus on percentile rankings of AC may overstate the salience of disparities. If differences in percentile rankings among subsets of urban residents in different locations correspond to only slight variations in heat exposure amplification, indicators of social vulnerability, or the probability of residential AC, or any combination thereof, then one might argue that there is little meaningful difference in adaptive capacity or residual vulnerability. However, given evidence that heat exposure increases the relative risk of a broad range of illnesses (7, 9, 70), a key question is whether—and if so, how—even seemingly small differences along these dimensions can translate into substantial differences in adverse health outcomes. Elucidating these issues is a priority for future research.

Notwithstanding these shortcomings, our findings highlight multiple disparities that amplify vulnerability to the negative effects of heat throughout a comprehensive sample of US metropolitan areas. Our spatially refined probabilities of residential AC prevalence reflect a number of regional and local climatic, socioeconomic, demographic, and infrastructural contexts, while the granularity of estimates allows us to gain insight into intra-urban differences in the spatial distribution of AC with broader implications for characterizing urban populations’ risk with regards to heat, informing heat resilience policies, and assessing the effect of heat adaptation on health outcomes. The distribution of AC is widely uneven both between and within US metropolitan areas and reinforce existing disparities in social vulnerability and surface UHI intensity. Our findings bolster evidence of the most vulnerable populations being disproportionately impacted by heat, in addition to bearing the additional burden of comparatively lower AC availability, challenging their ability to adapt to heat stress. Moreover, future analyses can combine these high-resolution estimates of residential AC with data on health outcomes to empirically determine the extent to which AC prevalence impacts heat vulnerability broadly, across urban populations, and at finer scales within cities.

## Materials and Methods

Our approach draws on empirical studies of the drivers of AC prevalence and adoption and the socioeconomic and demographic determinants of heat-related health impacts. Leveraging the methodological contribution by Gronlund and Berrocal (32), we specify a three-level multilevel mixed model of household presence of AC (i.e. either central or room AC) that we estimate using AHS microdata, and then apply our model to ACS data at the census tract level to predict probabilities of residential AC within a large sample of US cities. We interpret these probabilities as the proportion of prevalence of AC in a given census tract, which is used to calculate the percentile rankings of census tracts within each city and used to indicate metropolitan area-specific disparities in access to cooling. To characterize how this could affect the spatial distribution of heat health vulnerability, we assess how the latter correlates with indicators of social vulnerability and summer daytime SUHI intensity.

### Data

We use AHS public use microdata, stratified according to the demand for cooling. AHS metropolitan and national samples for 2003 to 2019 were combined, yielding 325,744 household observations within 115 core-based statistical areas (CBSAs, which collectively refer to metropolitan and micropolitan statistical areas delineated by the Office of Management and Budget—denoted “metropolitan areas” throughout the text). Each observation provided information on characteristics of the household’s residence as well as socioeconomic and demographic attributes of household respondents. We focused on variables in the AHS that were also recorded at the census tract level in the ACS 5-y estimates: survey year, metropolitan area of the household, tenure (rent or own), year built, presence of room or central AC, rent, market value, unit type (single or multifamily), number of bedrooms, income, age (<29, 30 to 49, 50 to 64, and >65), race (White, Black or African American, Asian, other race, two or more races), ethnicity (Hispanic or Latino), and educational attainment (no high school diploma, high school diploma, some college, bachelor’s degree, or higher).

Metropolitan areas were grouped into quintiles of annual cooling degree days (CDDs–the excess degrees of each day’s average temperature above 18°C, aggregated over the number of annual days with temperatures exceeding 18°C) tabulated at the county scale by the National Centers for Environmental Information (NCEI) Climate Divisional Database [NClimDiv– (71, 72)]. We matched metropolitan areas with their constituent metropolitan counties’ CDDs over the 1981 to 2010 period and computed area-weighted climatic annual average cooling degree days for each city (Supplementary Table S3).

ACS 5-y census tract estimates for 2015 to 2019 were combined with our fitted empirical model to construct fine spatial scale predictions of AC prevalence. The ACS is a nationally representative annual survey that records information on the social, economic, housing, and demographic characteristics of the population to produce 5-y estimates at various geographic aggregations—a US census tract is a geographic entity representing a statistical subdivision of a county (or county equivalent). We obtained census tract level estimates for our empirical model covariates (population, tenure, year built, median housing value, units in structure, number of bedrooms, median income, age, race, ethnicity, and education) for our sample of 115 metropolitan areas. ACS population equivalents of AHS household-level categorical variables (e.g. tenure, income, or race categories) were constructed as population shares (e.g. the proportion of the tract’s population representing a certain characteristic, such as owning a house or Hispanic or Latino ethnicity). For housing value and income, census tract median values were employed.

### Indicators of vulnerability

Within metropolitan areas, we assessed correlations between inter-tract disparities in residential AC prevalence and differences in high temperature exposure amplification and socioeconomic and demographic determinants of heat vulnerability. Tract-level population characteristics (race, ethnicity, age, education, and income) were obtained from ACS. Tract population shares of Black or African American race, Hispanic or Latino ethnicity, elderly (age 65 y and older), median income, and educational attainment (no high school diploma) are percentile ranked within their respective city and evaluated against their AC percentile ranking, grouped by quintile of metro area climatic average annual CDDs. The same applies for each tract’s overall and summary theme SVI score. For composite indicators of social vulnerability at the tract level we used the SVI (37, 73), a relative ranking of the vulnerability of locations based on 15 variables grouped into four thematic categories: (1) socioeconomic status (poverty, unemployment, income, and education); (2) household composition (age, disability, and single parenthood); (3) race, ethnicity, and language (minority and English language proficiency); and (4) housing and transportation (multiunit structures, mobiles homes, crowding, access to personal vehicle, and group quarters). Both the overall SVI and the four thematic SVIs are on 0 to 1 scales, with 1 indicating the highest social vulnerability (37). Since the SVI is provided as a national ranking of tract-level vulnerability and our analysis is only for a subset of US cities, we recalculated metropolitan area-specific SVI percentiles following the methodology used by CDC (37). This allowed for consistency with our metro area-scale AC prevalence percentiles and enabled us to similarly assess within-city variability in social vulnerability.

Amplification of exposure to extreme high temperatures is assessed using summer daytime values of SUHI, obtained from Chakraborty et al. (74). These data consist of clear-sky SUHI intensities for all urbanized areas in the US and their constituent census tracts, computed by combining several remotely sensed variables [land surface temperature from NASA’s Moderate Resolution Imaging Spectroradiometer (MODIS) 8-day and daily LST products, MODIS 8-day surface reflectance product, USGS’ Global Multi-Resolution Terrain Elevation Data (GMTED) and National Land Cover Database (NLCD) tree canopy dataset, as well as the European Space Agency’s Climate Change Initiative (ESA CCI) land cover data] on the footprint of Census-delineated urban areas [details in Chakraborty et al. (38)]. We extract annual and summer daytime SUHI intensities for census tracts corresponding to the 115 metro areas in our sample. Due to differences between urbanized areas and metropolitan area geographic boundaries, SUHI values are not available for a small fraction (7.5%) of the census tracts in our full sample. Since SUHI is based upon differences in urban-rural land surface temperatures (*T*_s_), we acknowledge that it is an imperfect measure of heat exposure [the relevance of remotely-sensed land surface temperatures as a measure of urban heat stress is discussed in detail elsewhere (65, 66, 76)]. Briefly, the relationship between air temperatures (*T*a) and *T*_s_ is complex, especially at fine spatial (intra-urban) and temporal (hourly, daily) scales. Moreover, heat stress is influenced not only by temperature, but by a myriad of inputs pertaining to urban climate processes such as humidity, solar radiation, wind speed, and tree canopy/shading (35). As such, we more aptly regard tract values of SUHI intensity as an indicator of potential exposure *amplification*.

### Empirical approach

For our empirical approach, we employ a three-level hierarchical specification that models households (indexed by *h*) nested within CBSAs (indexed by *i*) nested within climatic CDD quintiles (indexed by *j*). Our dependent variable is a binary indicator for the presence of AC, in households within cities within climate zones, $A{C}_{h,i,j\ }$, while our covariates are housing characteristics, ${\boldsymbol{X}}_{h,i,j}^{Struct}$, (unit type, tenure, number of bedrooms, and real market value) and characteristics of their constituent households, ${\boldsymbol{X}}_{h,i,j}^{Hhold}$––both socioeconomic (income, educational attainment), and demographic (age, race, and ethnicity)—all observed across different AHS survey waves (indexed by *y*). The model is written as:

(1) $$\begin{eqnarray*}
logit\ \left( {\mathbb{E}\left[ {A{C}_{h,i,j}\left( y \right)} \right]} \right) &=& \ \alpha + {\boldsymbol{X}}_{h,i,j}^{Struct}\left( y \right){\boldsymbol{\beta }} + {\boldsymbol{X}}_{h,i,j}^{Hhold}\left( y \right){\boldsymbol{\gamma }} \nonumber \\
&& + \ \vartheta _j^* + \mu _{i,j}^* + \tau _{i,j}^*y
,
\end{eqnarray*}
$$

in which $\alpha$ indicates the conditional mean propensity of household presence of AC, $\vartheta _j^{\rm{*}}$ is a random intercept for CDD quintiles that controls for the effect for unobserved time-invariant climatic influences, $\mu _{i,j}^{\rm{*}}$ is a random intercept for cities that controls for unobserved time-invariant CBSA-level shocks, and $\tau _{i,j}^{\rm{*}}y$ is a random city-specific time trend that controls for shifts in the conditional mean probability across cities and years in response to unobserved secular forces associated with urban growth and change. The parameters of interest, $\beta$ and $\gamma$, are deterministic (i.e. not random slopes) coefficient vectors that capture the average effects on AC ownership of structural and household (socioeconomic and demographic) characteristics across cities and CDD quintiles.

Equation 1’s advance is to incorporate the socioeconomic and demographic predictors that allow us to draw the inferences that constitute our main results. Its foundation is Gronlund and Berrocal’s (32) model of AC prevalence as a function of housing attributes and the regional climates in which houses are located. (32) That analysis specifies a two-level model (households within CBSAs) that introduces CDDs as a continuous modifier of the effect of structure characteristics through multiple interactions:

(2) $$\begin{eqnarray*}
&& logit{\rm{\ }}\left( {\mathbb{E}\left[ {A{C}_{h,i}\left( y \right)} \right]} \right) = {\rm{\ }}\alpha + {\boldsymbol{X}}_{h,i}^{Struct}\left( y \right){{\boldsymbol{\beta }}}_1 + \left( {CD{D}_i \cdot {\boldsymbol{X}}_{h,i}^{Struct}\left( y \right)} \right){{\boldsymbol{\beta }}}_2 \nonumber \\
&& \quad + \ {\delta }_1CD{D}_i + {\delta }_2y + {\delta }_3\left( {CD{D}_i \cdot y} \right) + {\delta }_5\left( {Tenur{e}_{h,i}\left( y \right) \cdot y} \right) \nonumber \\
&& \quad + \ {\delta }_6\left( {CD{D}_i \cdot Tenur{e}_{h,i}\left( y \right) \cdot y} \right) + {\bar{\mu }}_i
.
\end{eqnarray*}
$$

Equation 1 thus captures the mean influence of the continuously-varying effect of housing characteristics in Eq. 2: ${\boldsymbol{\beta }} \approx \mathbb{E}( {{{\boldsymbol{\beta }}}_1 + {{\boldsymbol{\beta }}}_2 \cdot CD{D}_i} )$, while controlling for city- and year-varying unobserved shocks whose impacts potentially vary with cities’ climate: $\vartheta _j^{\rm{*}} + \mu _{i,j}^{\rm{*}} + \tau _{i,j}^{\rm{*}}y \approx {\delta }_1CD{D}_i + {\delta }_2y + {\delta }_3( {CD{D}_i \cdot y} ) + {\bar{\mu }}_i$. The benefit of stratifying by CDD quintile is enabling variation in the probability of residential AC to be conditioned on a much broader slate of predictors while avoiding the multicollinearity associated with multiple interaction terms.

Using *t* to index census tracts, we apply our fitted Eq. 1 to ACS tract-level predictors, ${{\boldsymbol{\tilde{X}}}}_{t,i,j}$, for the 115 cities in our sample over the 2015–2019 period ($\tilde{y}$). The result is our predicted AC prevalence:

(3) \begin{equation*} {\rm{\ }}{P}_{t,i,j} = {\boldsymbol{\tilde{X}}}_{t,i,j}^{Struct}\ {\boldsymbol{\hat{\beta }}} + {\boldsymbol{\tilde{X}}}_{t,i,j}^{Hhold}{\boldsymbol{\hat{\gamma }}} + \hat{\vartheta }_j^* + \hat{\mu }_{i,j}^* + \hat{\tau }_{i,j}^*\tilde{y}. \end{equation*}

We computed average AC prevalence for metropolitan areas as the population-weighted average of probabilities for tracts within their encompassing CBSAs. Scatterplots of metropolitan area-level (central) AC and (any) AC prevalence estimates are compared to AHS in Supplementary Fig. S7b. (See Supplementary Material for details, robustness checks, validation of predicted probabilities, and comparison with alternative empirical specifications, Supplementary Tables S1 and S2.) Analyses were conducted in R (version 4.0.5), and estimation procedures used a generalized linear mixed-effects modeling package (lme4, version 1.1.27.1).

## Supplementary Material

pgac210_Supplemental_File

## Data Availability

Output AC prevalence data are publicly available at Harvard Dataverse: https://doi.org/10.7910/DVN/HWFVP6. Input data used in this analysis are publicly available at the following: AHS microdata: https://www.census.gov/programs-surveys/ahs/data.html; 2015–2019 5-y ACS: https://api.census.gov/data/2019/acs/acs5/variables.html; CDDs from NClimDiv https://www.ncei.noaa.gov/pub/data/cirs/climdiv/; CDC 2018 census tract SVI: https://www.atsdr.cdc.gov/placeandhealth/svi/data_documentation_download.html; and census tract summer daytime SUHI: https://data.mendeley.com/datasets/x9mv4krnm2/2.
