# Supplementary material for: Inequality in the availability of residential air conditioning across 115 US metropolitan areas
Source: PNAS Nexus. 2022 Sep 24;1(4):pgac210. doi: 10.1093/pnasnexus/pgac210 (PMC9802221; doi:10.1093/pnasnexus/pgac210)
Supplement: pgac210_Supplemental_File [file pgac210_supplemental_file.docx]

**
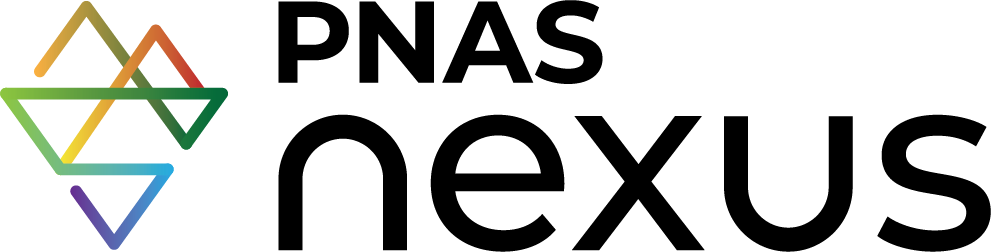
**

**Supplementary Information for**

Inequality in the availability of residential air conditioning across 115 US metropolitan areas

Yasmin Romitti, Ian Sue Wing, Keith Spangler, Gregory A. Wellenius

*Yasmin Romitti, 685 Commonwealth Avenue, Boston, MA 02215

**Email:**  [yromitti@bu.edu](mailto:yromitti@bu.edu)

**This PDF file includes:**

Supplementary text

Figures S1 to S7

Tables S1 to S5

SI References

**Supplementary Information Text**

**Robustness and Validation**

We explore the robustness of our approach through several alternate specifications, including:

A random intercept model for CBSAs with fixed effect covariates that vary continuously with CDD exposure;

$$\begin{aligned} logit\left( \mathbb{E}\left[ {AC}_{h,i,j} \right] \right)=(\alpha^{0}+\alpha^{1}CDD)+\boldsymbol{X}_{h,i,j}^{Struct}(\boldsymbol{\beta}^{0}\boldsymbol{+}\boldsymbol{\beta}^{1}CDD) +\boldsymbol{X}_{h,i,j}^{Hhold}(\boldsymbol{\gamma}^{0}\boldsymbol{+}\boldsymbol{\gamma}^{1}CDD)+\mu_{i,j}+\tau_{i,j}T_{i,j}\#\left( A1 \right) \end{aligned}$$

a random intercept model for CBSAs with fixed effect covariates stratified by CDD quintile;

$$\begin{aligned} logit\left( \mathbb{E}\left[ {AC}_{h,i,j} \right] \right)=\alpha+\boldsymbol{X}_{h,i,j}^{Struct}\boldsymbol{\beta}_{j}+\boldsymbol{X}_{h,i,j}^{Hhold}\boldsymbol{\gamma}_{j}+\mu_{i,j}+\tau_{i,j}T_{i,j}\#\left( A2 \right) \end{aligned}$$

and a random intercept model for CBSAs with random slopes for CDD quintile;

$$\begin{aligned} logit\left( \mathbb{E}\left[ {AC}_{h,i,j} \right] \right)=\alpha+\boldsymbol{X}_{h,i,j}^{Struct}\boldsymbol{\beta}+\boldsymbol{X}_{h,i,j}^{Hhold}\boldsymbol{\gamma}+\mu_{i,j}+\tau_{i,j}{CDD}_{i,j}^{quintile}\#\left( A3 \right) \end{aligned}$$

Across models A1-A3, the direction and magnitude of the coefficients on main covariates remain similar to that of our primary specification, with the largest magnitude of difference found in the intercept (the average log odds of AC, all else equal) and random (CBSA-specific) intercepts. Models A1 and A2 both include interactions for CDDs with each of our main covariates: for both specifications, time trend, unit type, homeownership, and real market value modified by CDD quintile or exposure are significant housing predictors of AC prevalence, while this modification for all age groups, Black or African American and Asian race, and educational attainment [some college and bachelor’s degree or higher] were significant socioeconomic and demographic predictors. Real income and building age modified by climate were only found to be significant in A2—when stratified by CDD quintile as opposed to the climate average annual number of CDDs (A1). The largest differences in the values of random effects were found in specification 1c versus our primary specification and A1-A2, as might be expected by the inclusion of CDD quintile as a random slope rather than as a modification effect.

Separately, we apply the structure of our primary specification that estimates the presence of *any* household AC (mANY) to additionally estimate models of central AC (mCE) and room AC (mRM). Quintile of CDD explains the majority of variance (64%) among groups for central AC, whereas CBSA accounts for more variance among groups for room AC (72%). Across our two main types of correlates (housing characteristics and sociodemographic characteristics), almost all socioeconomic and demographic attributes (represented by dummy variables, except real income) are significant predictors of central AC, while fewer (namely, age [30 to 49], Black or African American race, Asian race, and educational attainment [high school diploma, some college]) are significant determinants of room AC. Across all models, real market value and number of bedrooms markedly increase the log odds of AC presence. Meanwhile, building attributes such as age, tenure (rent or own) and unit type (single or multifamily) were inversely associated across mCE and mRM—the resulting coefficients in mANY are subsequently similar in sign but smaller in magnitude than mCE.

We assess the predictive power of our model and each alternate specification using a hold-out validation procedure. Each model is trained using 80% of our AHS estimation sample, holding-out the remaining 20% in order to compare to our predicted probabilities. We evaluate model performance by using receiver operating characteristic (ROC) curves to compute area under the curve (AUC) metrics, as well as sensitivity (true positive rate), and specificity (true negative rate) values using several classification thresholds, Table S1. We classify our probabilities using the same threshold values as in Gronlund and Berrocal (2020), 0.5 and 0.9, and further test an additional cut-off of 0.8, the threshold which maximizes sensitivity without forgoing too large a fraction of specificity. At a threshold of 0.5, our primary specification (1) correctly identifies households with any type of AC 97.6% of the time at the expense of only correctly identifying households without AC 42.7% of the time, with an overall AUC value of 90.2%. Higher threshold values result in higher specificity but lower sensitivity: using a cut-off value of 0.8, sensitivity drops to 89.2%, but sensitivity increases to 72.4%.Importantly, our primary model performs similarly to alternate models A1-A3, with no differences greater than 0.006 for any metric.

**Table S1** Sensitivity, specificity, and AUC for primary and alternate specifications of any AC mixed logistic models computed using hold-out validation.

| **Model** | **Threshold** | **Sensitivity** | **Specificity** | **AUC** |
| --- | --- | --- | --- | --- |
| Three-level multilevel mixed logistic (1) | 0.5 | 0.9755 | 0.4270 | 0.9016 |
|  | 0.8 | 0.8917 | 0.7237 |  |
|  | 0.9 | 0.7864 | 0.8608 |  |
| Random intercept for CBSAs with covariates*continuous CDD (A1) | 0.5 | 0.9755 | 0.4298 | 0.9023 |
|  | 0.8 | 0.8921 | 0.7244 |  |
|  | 0.9 | 0.7872 | 0.8609 |  |
| Random intercept for CBSAs with covariates*CDD quintile (A2) | 0.5 | 0.9751 | 0.4329 | 0.9028 |
|  | 0.8 | 0.8899 | 0.7253 |  |
|  | 0.9 | 0.7877 | 0.8593 |  |
| Random intercept for CBSAs with CDD quintile as random slopes (A3) | 0.5 | 0.9751 | 0.4311 | 0.9008 |
|  | 0.8 | 0.8901 | 0.7278 |  |
|  | 0.9 | 0.7866 | 0.8583 |  |

*AHS benchmarking*

Finally, we benchmark our out-of-sample predictions against AHS. We aggregate our out-of-sample tract-level probabilities to metro area-level predicted probabilities of any AC and compare these to metropolitan area-level weighted (using AHS-provided survey weights) multi-year means of any AC from AHS for our sample, thus precluding a strict percentage-to-percentage comparison. Nonetheless, interpreting tract probabilities as reflective of proportion of AC prevalence, we find that 78% of metropolitan area-level estimates are within 5 percentage points of AHS (90 cities), while 89% are within 10 percentage points of AHS (102 cities), Fig. S1. Metro areas whose aggregate estimates are outside this threshold are primarily those in CDD quintiles 1 and 2, and one in quintile 3 (San Diego, CA). Our empirical model tends to overpredict the probability of AC across census tracts, a phenomenon we speculate stems from variations in the household presence of room AC depending on regional climate and ownership of window units. Alternate specifications (A1-A3) perform similarly to our primary model when benchmarked against CBSA-level AHS values, with a mean absolute percentage error (MAPE) ranging from 6.6-7.9%, Table S2. These alternate specifications also exhibit the same pattern of MAPE when comparisons are done stratified by CDD quintile, with the largest differences found in CDD quintile 1 and the smallest in CDD quintiles 4 and 5. Mean and standard deviations of tract level probabilities for all four models are shown in Table S3, along with CDDs for each CBSA.

**Table S2** Mean absolute percentage error of metropolitan area-level predicted probabilities of residential (any) AC from all model specifications compared to metropolitan area-level averages from AHS by CBSA CDD quintiles and all CBSAs

| **Model** | **Quintile 1** | **Quintile 2** | **Quintile 3** | **Quintile 4** | **Quintile 5** | **All CBSAs** |
| --- | --- | --- | --- | --- | --- | --- |
| 1 | 28.2% | 6.3% | 3.1% | 0.8% | 1% | 7.9% |
| A1 | 29.1% | 6.3% | 3.1% | 0.8% | 0.9% | 7.9% |
| A2 | 27.4% | 6.4% | 3% | 0.9% | 0.9% | 7.7% |
| A3 | 21.4% | 6.5% | 3.2% | 1% | 1.1% | 6.6% |

*Inter- and intra-urban variation in residential AC*

Prior research has documented inter-urban variation in residential AC prevalence (e.g., O’Neill et al, 2005; Sera et al, 2020). We choose to compare our estimates to those shown in Sera et al. (2020) due to both the large sample of cities and use of similar data sources (i.e., AHS), Supplementary Fig. S5. Aggregated population-weighted metropolitan area-level predicted probabilities are substantially higher than the average central AC prevalence found by Sera et al. (2020). While a portion of this considerable difference is due to the units of comparison—aggregated predicted probabilities against percentages of prevalence—across study areas, this variation is additionally attributable to separate methodologies. Sera et al (2020) focus on central AC, in contrast to any AC, and reconstruct measures of AC over the time period 1973-2006 by smoothing irregular survey data from the U.S. Census of Population, AHS, and the US Energy Information Administration, Office of Energy Consumption Residential Energy Consumption Survey using a linear mixed effects model (see Supplementary Information for Sera et al, 2020 for additional details).

In addition, intra-urban variation in availability of residential AC has been explored across a subset of one or a few cities, most often in the context of broader analyses (i.e., assessments of heat vulnerability) and through the use property-level information (i.e., tax assessor databases). As described in the main text (see Discussion), data limitations prevent us from conducting head-to-head comparisons (apart from Gronlund and Berrocal, 2020). However, we do qualitatively evaluate the pattern of prevalence evidenced in prior studies compared to our predicted probabilities. Predicted probabilities of tract any AC in Detroit are on average 19.4% lower in Gronlund and Berrocal (2020) than our estimates for the city, Supplementary Fig. S6. We predict a median tract probability of AC of 0.9, in contrast to 0.75 in Gronlund and Berrocal (2020) with an overall range of 0.85 to 0.96 (compared to 0.62 to 0.97). These differences may in part reflect the aggregation of parcel predictions to the tract level by Gronlund and Berrocal (2020), but more likely highlight the influence of sociodemographic correlates that are included in our model specification. Further, using the model coefficients for central AC provided in Table 3 of Gronlund and Berrocal (2020) in conjunction with random effect coefficients shown in their Supplementary Table 1, we generate census tract probabilities of central AC for our sample metropolitan areas. Differences between tract probabilities for central AC compared to our estimates of tract any AC are greatest (on average differing by > 100%) in CDD quintile 1 (coolest climates) and subsequently decrease across warmer climates, with the smallest differences found in CDD quintiles 4 and 5. We further aggregate tract-level probabilities to generate population-weighted metropolitan area estimates, Supplementary Fig. S7, with which to benchmark Gronlund and Berrocal (2020) against AHS in addition to benchmarking our predictions against AHS. Generally, our estimates of prevalence are for the most part higher than those predicted using Gronlund and Berrocal (2020), as well as AHS—a divergence that is again more pronounced for CDD quintiles 1 and 2. This indicates that our model tends to overestimate residential AC. Mean absolute percentage deviation from AHS for estimates generated using Gronlund and Berrocal (2020) is 14.8%, with their model largely understating the presence of AC. In comparison, our model performs slightly better, with a mean absolute percentage deviation of 7.9%. While the distinction between central and any AC may account for a large portion of this discrepancy for cities in the lowest CDD quintiles, we suggest that the addition of correlates capturing socioeconomic and demographic factors in our empirical specification is the largest determinant of these differences, which decrease in very warm metro areas that tend to have high prevalence of AC.

A combination of property-level data obtained from tax assessors and AHS are used to compute prevalence of central AC across Los Angeles (LA metro area) and Maricopa (Phoenix metro area) counties in Fraser et al. (2017). Prevalence of central AC is shown to be much higher in Phoenix relative to LA, where on average 95% of households have central AC (in contrast to < 50% in LA). Our findings similarly reflect lower probabilities across census tracts in LA County compared to those in Maricopa County (median predicted probability of 0.99 versus 0.80). The high probabilities of AC we estimate across tracts in LA County contrasts the relatively low percentage of central AC documented in Fraser et al. (2017), a consequence of both the focus on central versus any AC and the use of tax assessor information for single family homes and small multi-family buildings alongside AHS household information for the remainder of residential buildings in LA. Similarly, Guirguis et al. (2018) illustrate differences in central AC across San Diego County stratified by climate zones. While Guirguis et al. (2018) demonstrate that only up to 40% of coastal residents have central AC, our predicted probability of any AC ranges from 0.47 to 0.89 across San Diego census tracts, with a median predicted probability of 0.71. However, we similarly find lower percentile rankings of AC along some coastal San Diego County tracts compared to the rest of the metropolitan area. Here we attribute contrasting patterns of prevalence to underlying data sources: while we employ ACS estimates for census tract predictions, Guirguis et al. utilize microdata from the 2009 California Residential Appliance Saturation Survey (KEMA, 2010). Ahn et al. (2022) utilize property information from the real estate database Estaded to assess spatial clustering of AC in Florida counties, reporting that most had over 75% of any type of AC. Across the 10 Florida metropolitan areas in our sample, we find a high predicted probability of AC, 0.99. Ahn et al. (2022) additionally assess intra-urban variation AC in Jacksonville, FL, finding little to no AC availability in census tracts located in the urban core, a spatial pattern that we corroborate across our sample of U.S. metro areas. Finally, we evaluate patterns of predicted AC prevalence in New York City counties against percentages provided in the New York City (NYC) 2017 Housing and Vacancy Survey (HVS) (U.S. Census Bureau; Gamarro et al, 2020). Average household AC prevalence is high across the city, at 91.6%, in broad agreement with our estimates: median predicted probability of 0.97 across NYC boroughs.

**Figure S1** Population-weighted metropolitan area-level predicted probabilities of residential AC compared to weighted AC prevalence from the American Housing Survey (AHS). Black line denotes 1:1 areas of agreement.

**
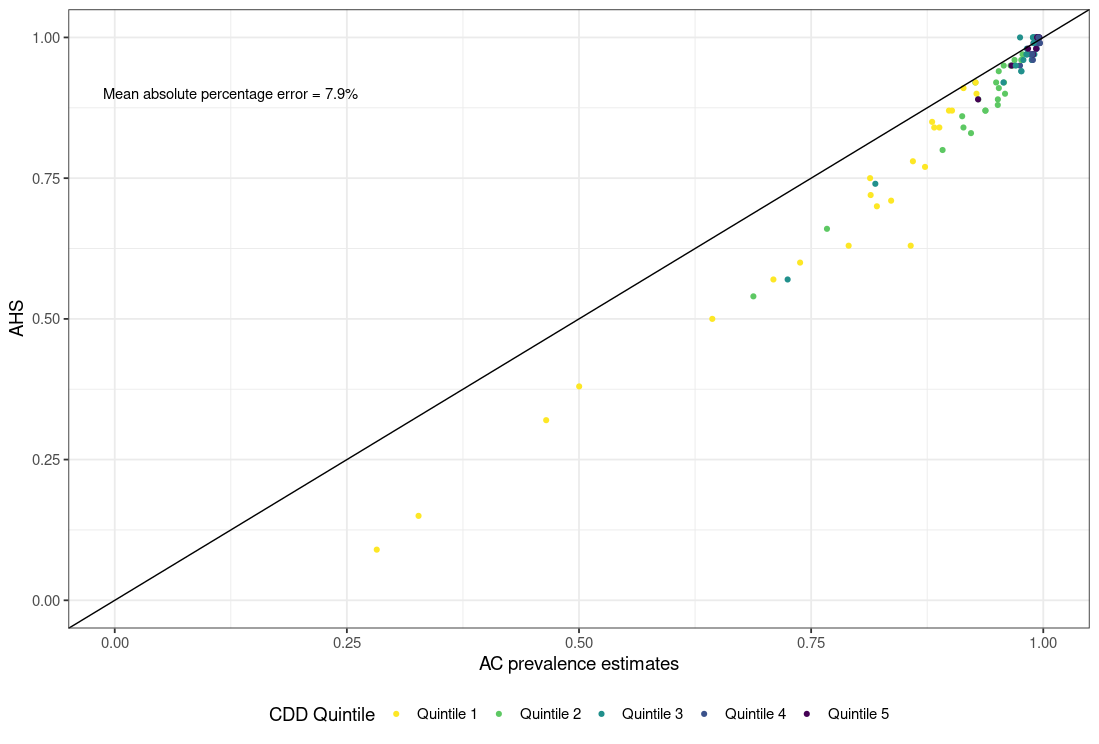
**


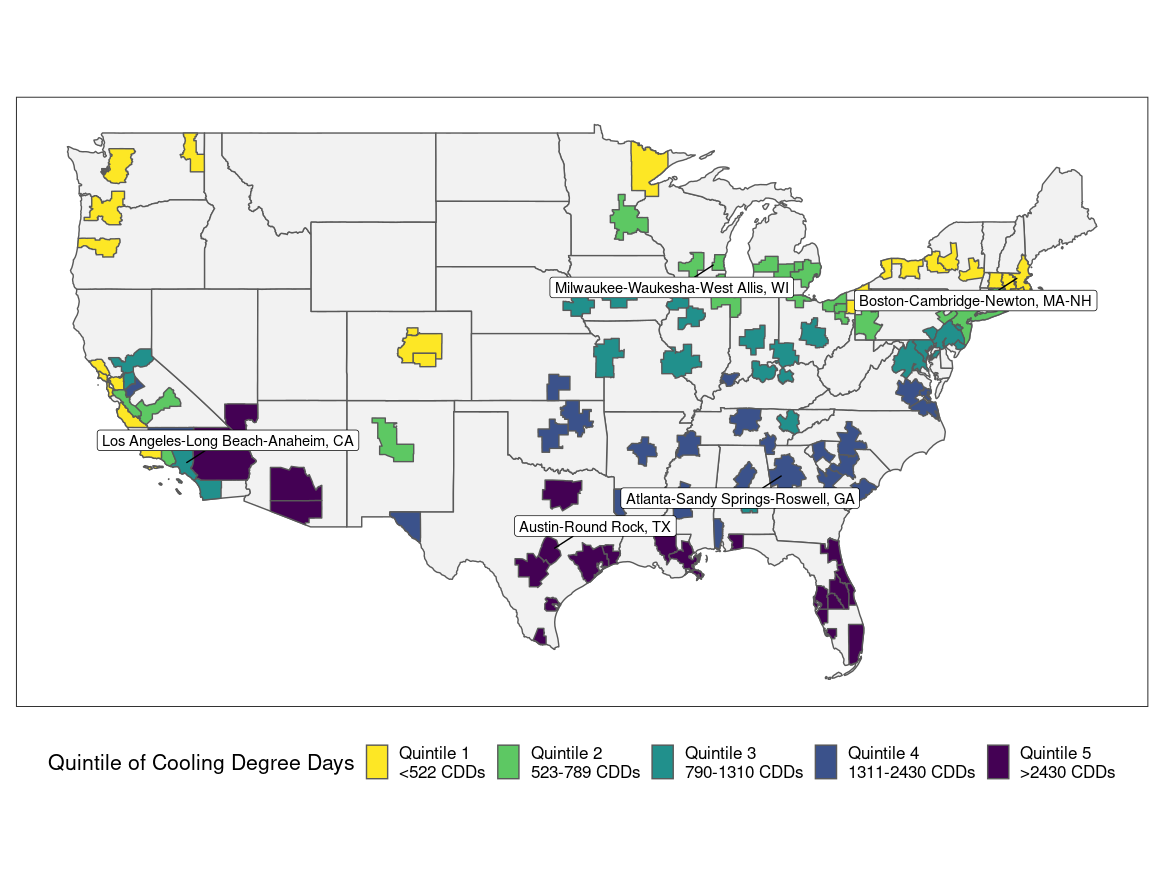
**Figure S2** Geographical distribution of sample metropolitan areas. Metropolitan area color corresponds to quintile of average annual cooling degree days (CDDs), labels indicate five representative cities from each CDD quintile.

**Figure S3** Intra-urban variation in percentile ranking of residential AC predicted probabilities in remaining 110 metropolitan areas. Pink dots indicate the downtown or financial center of the metro area.


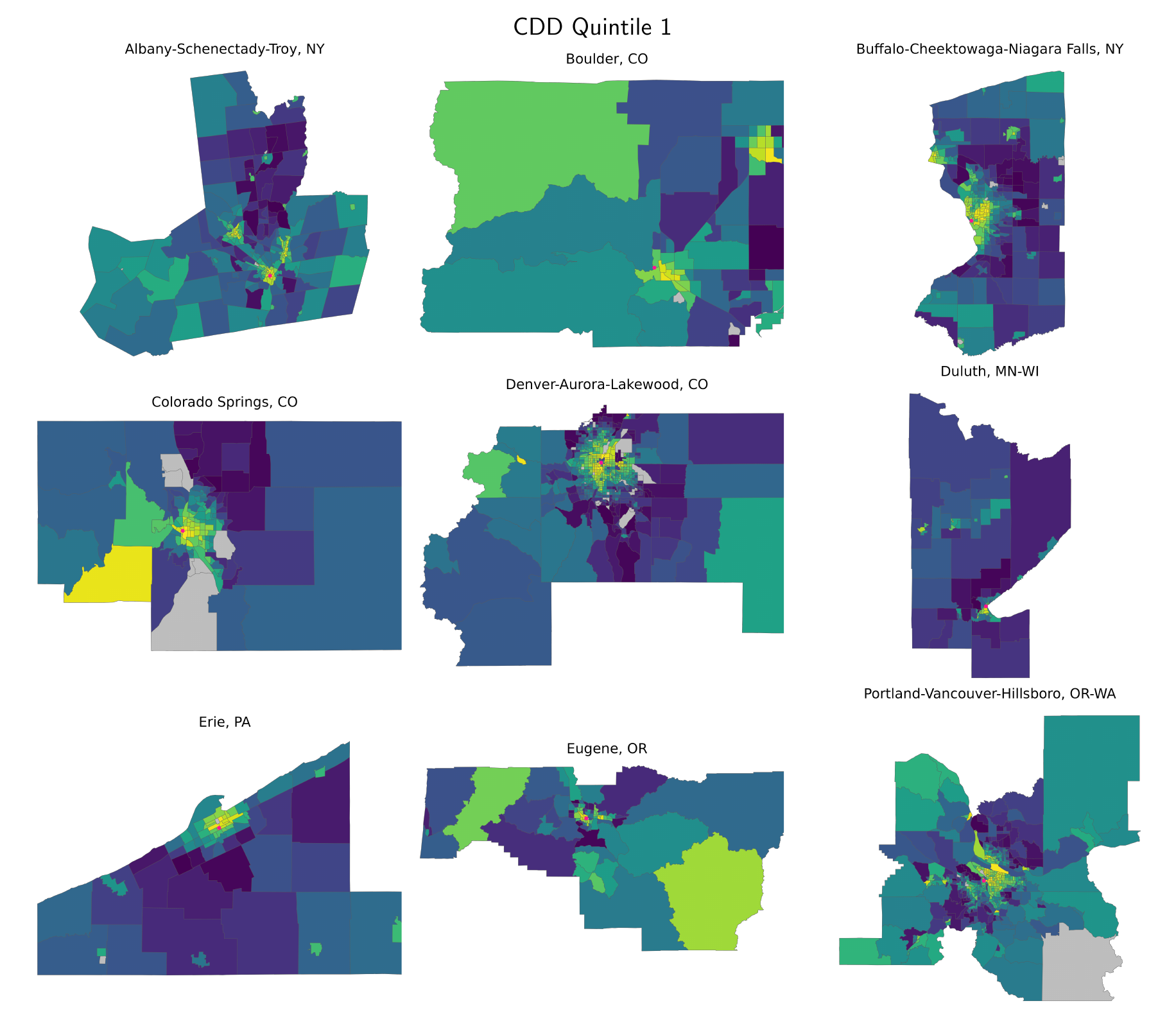

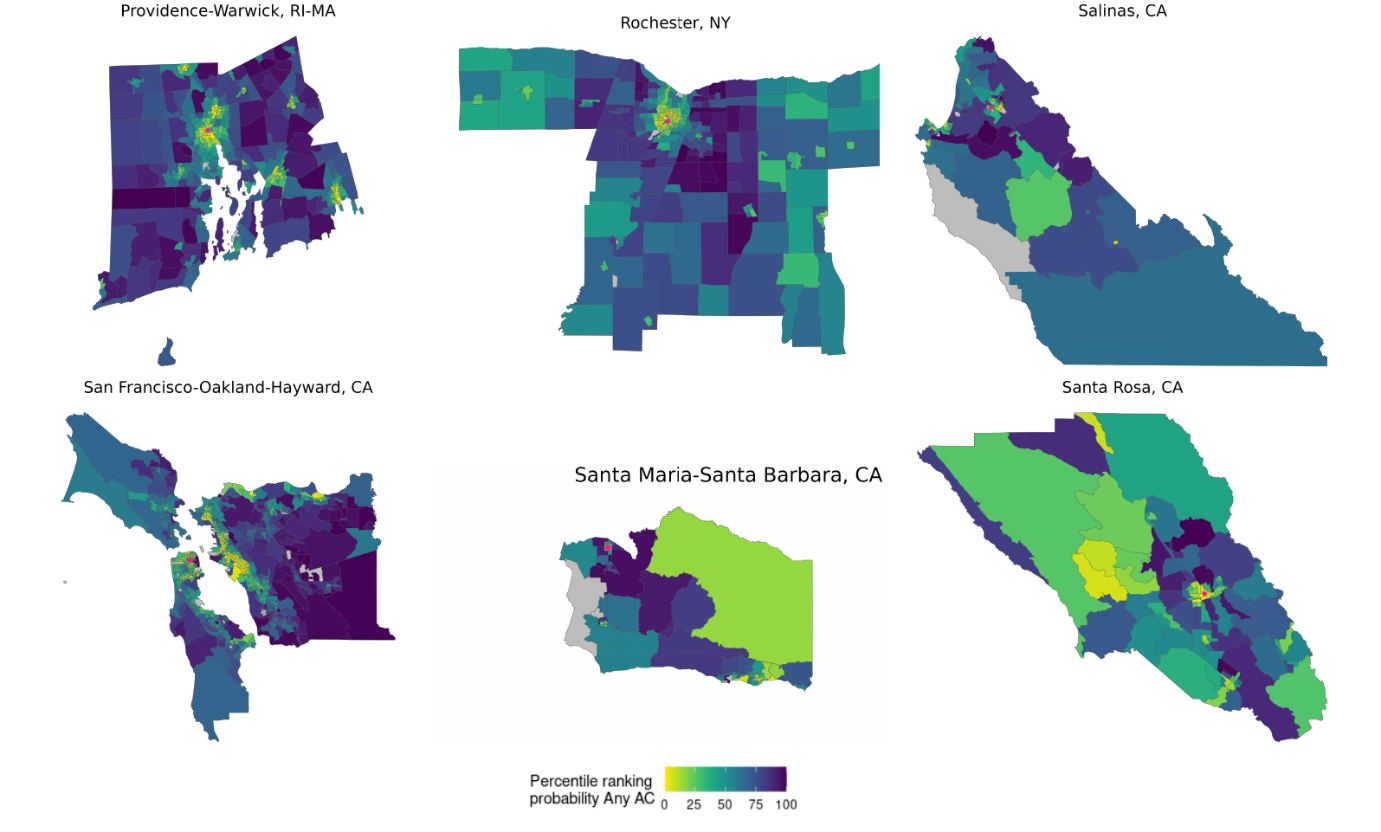

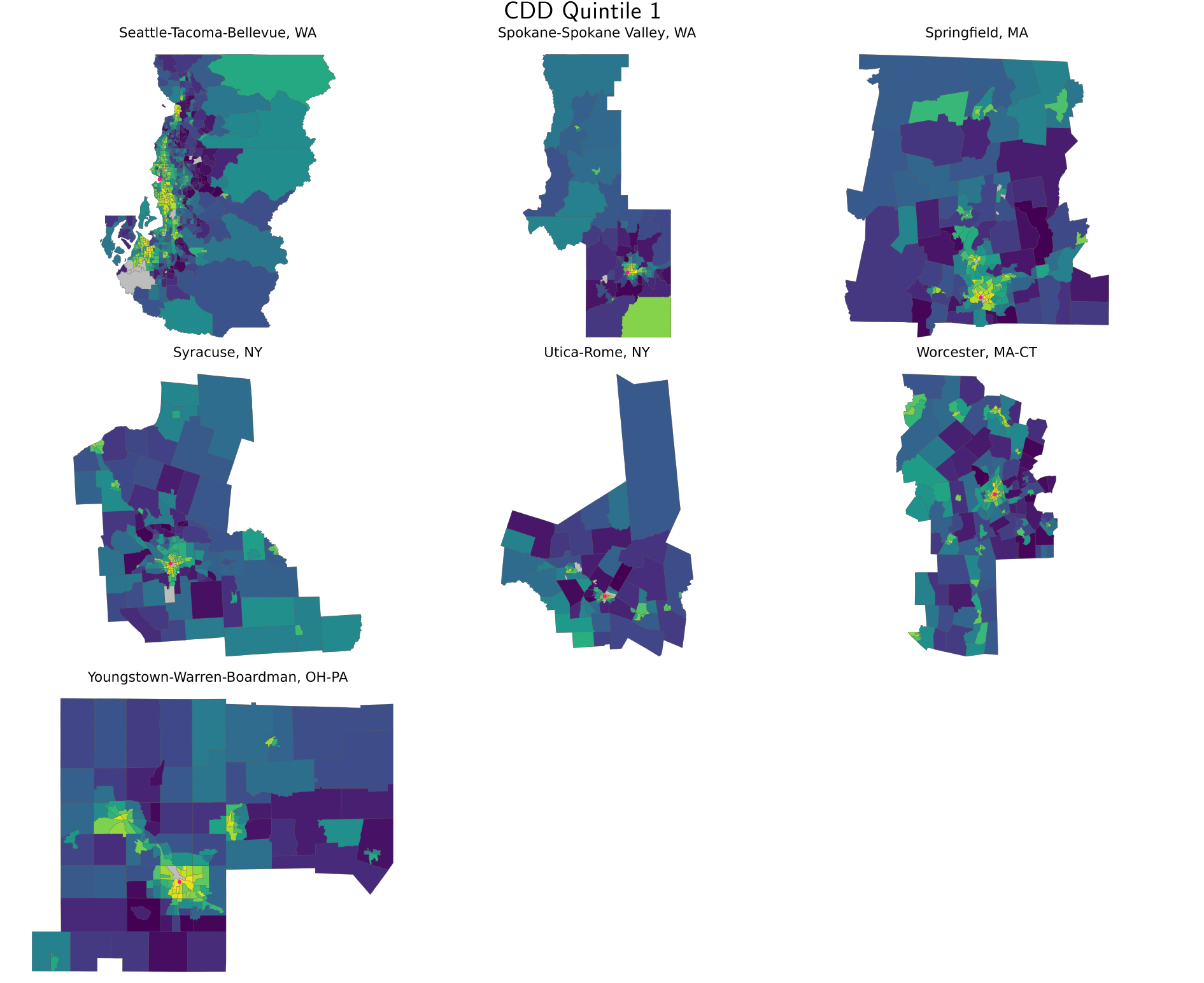

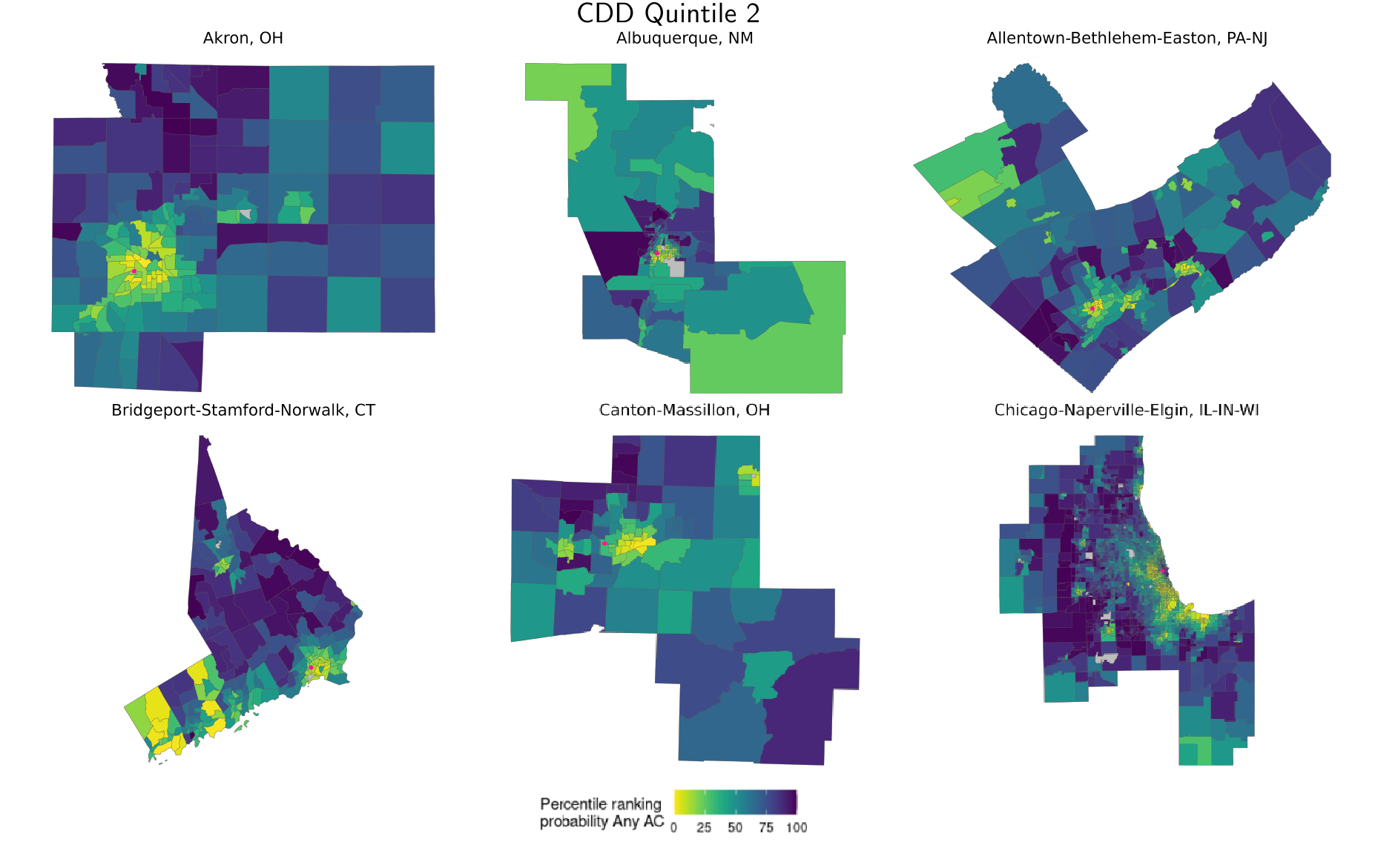

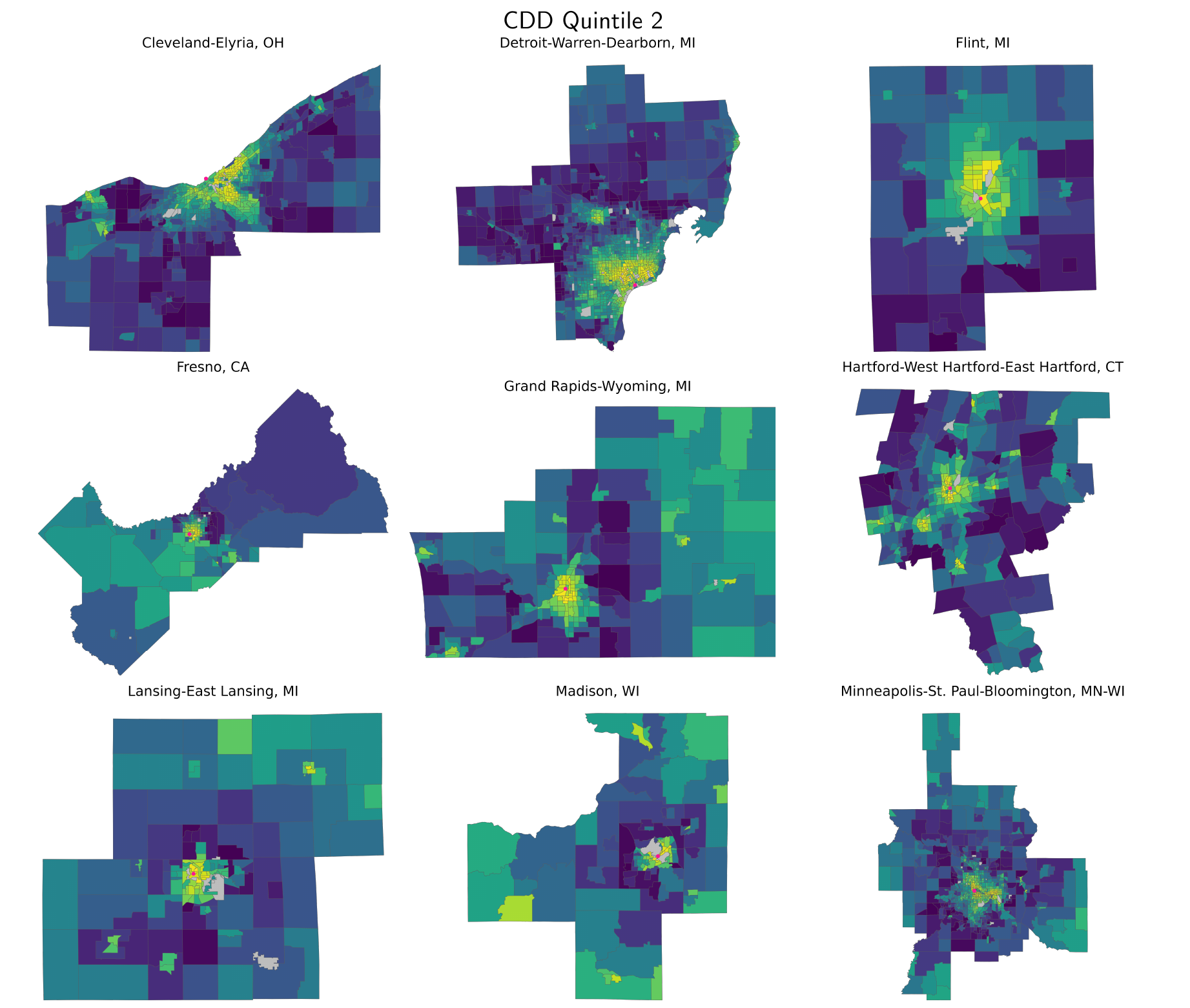

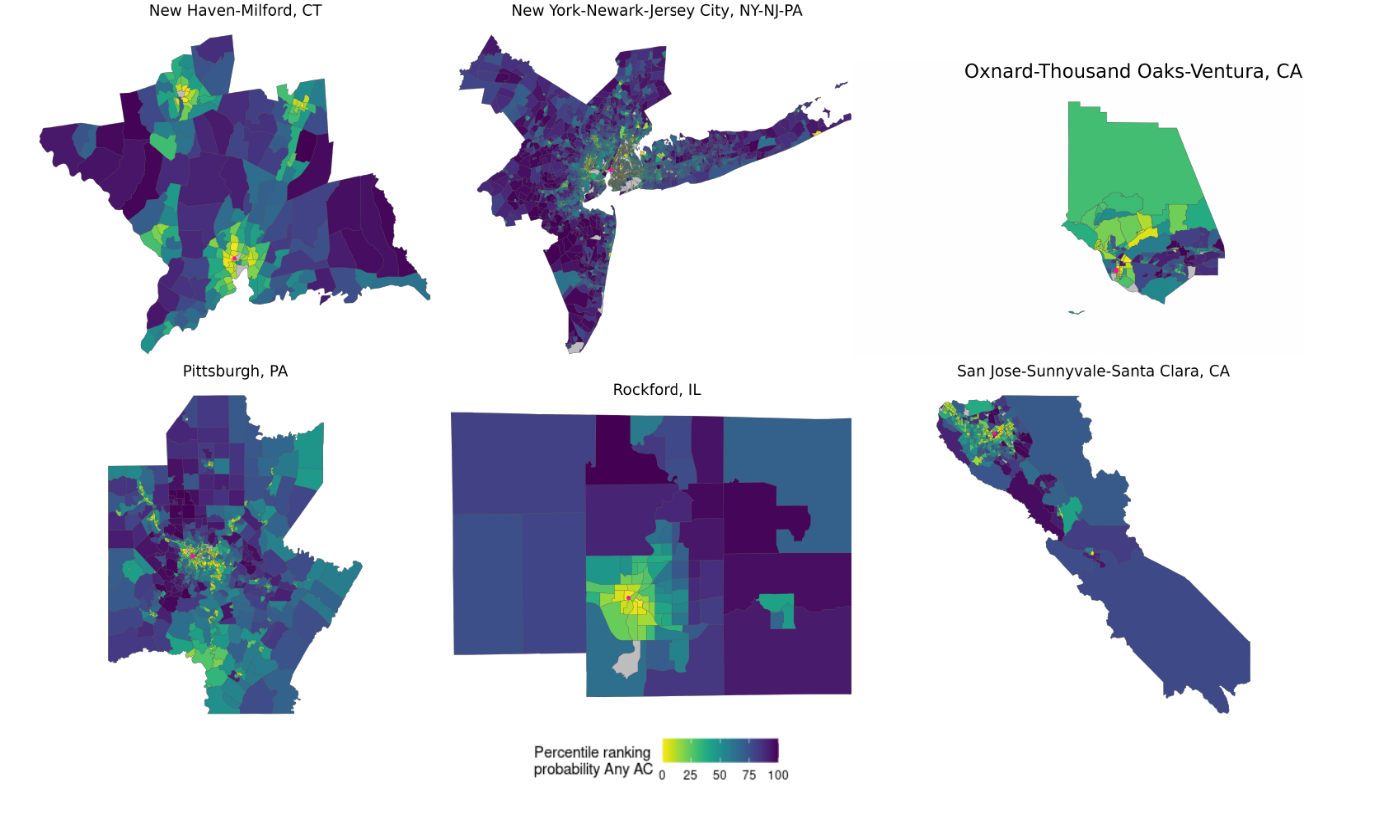

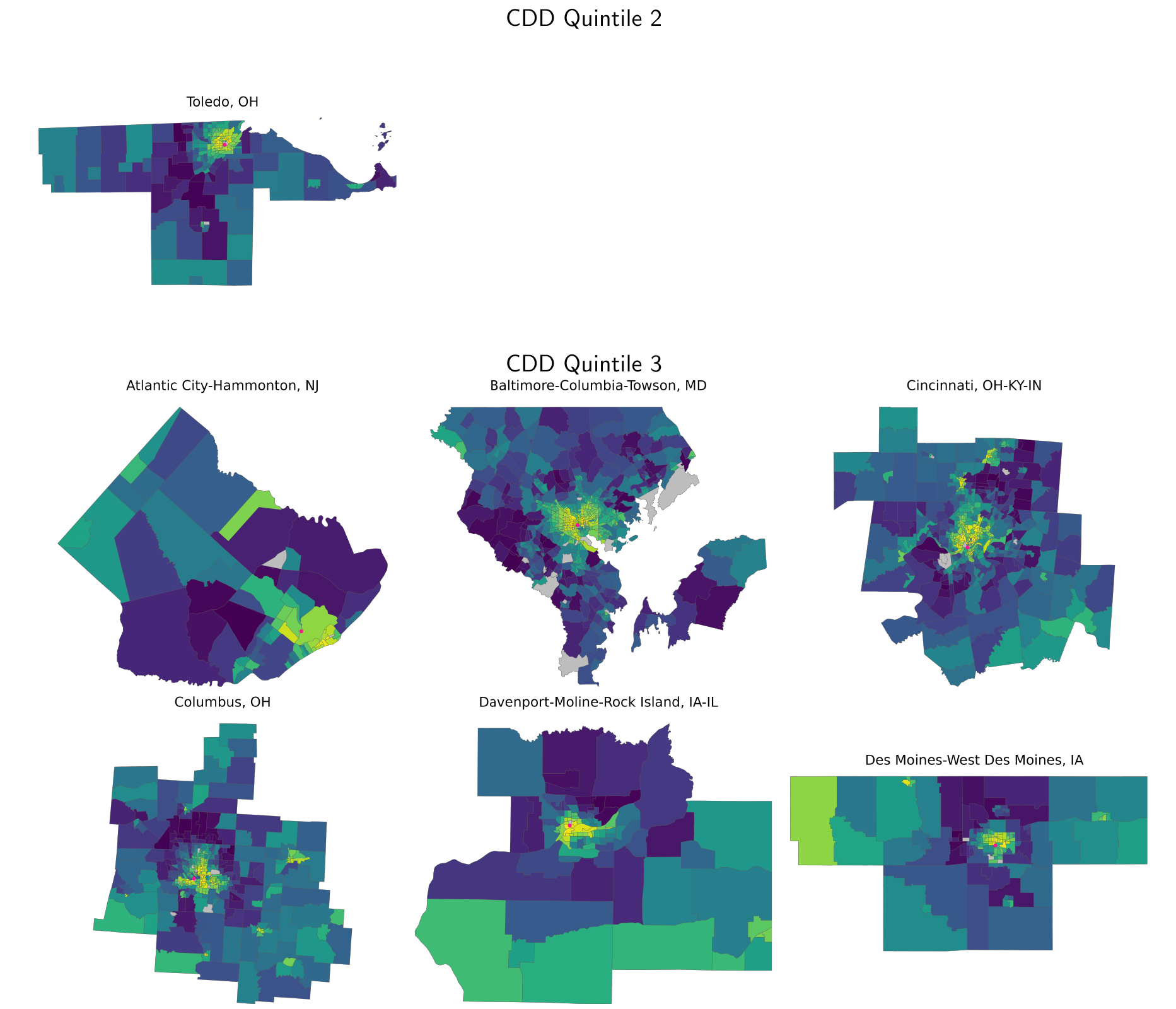

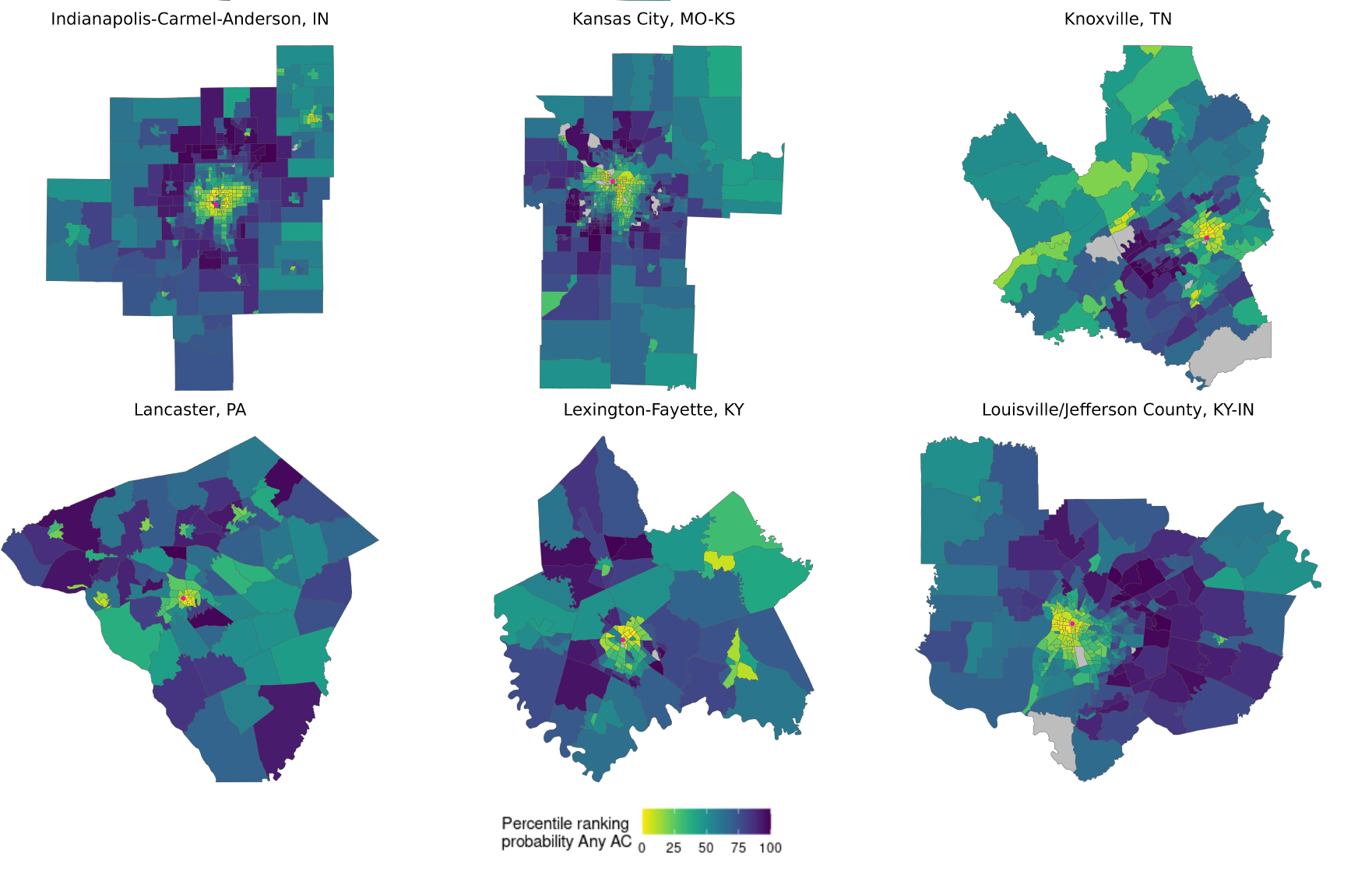

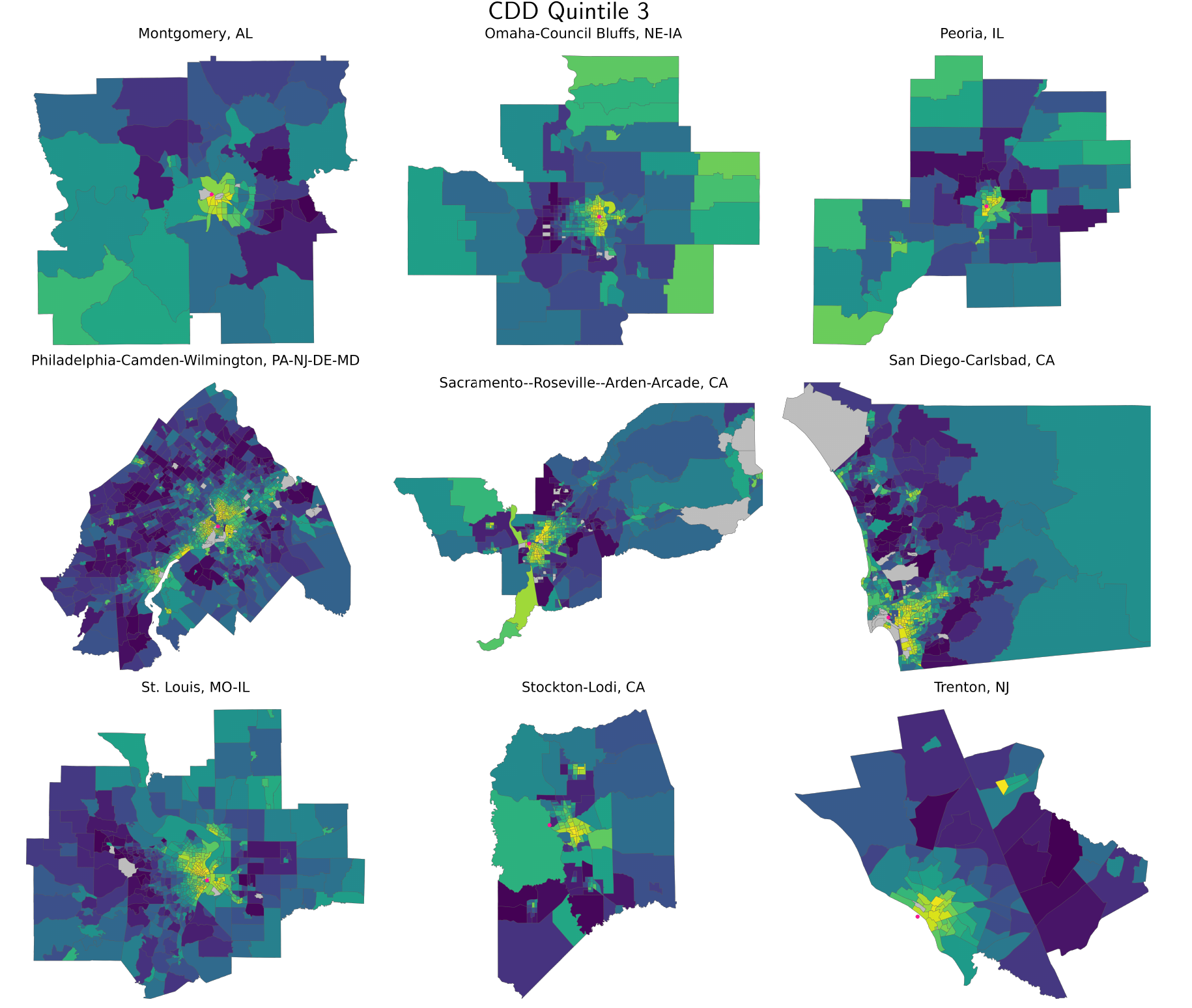

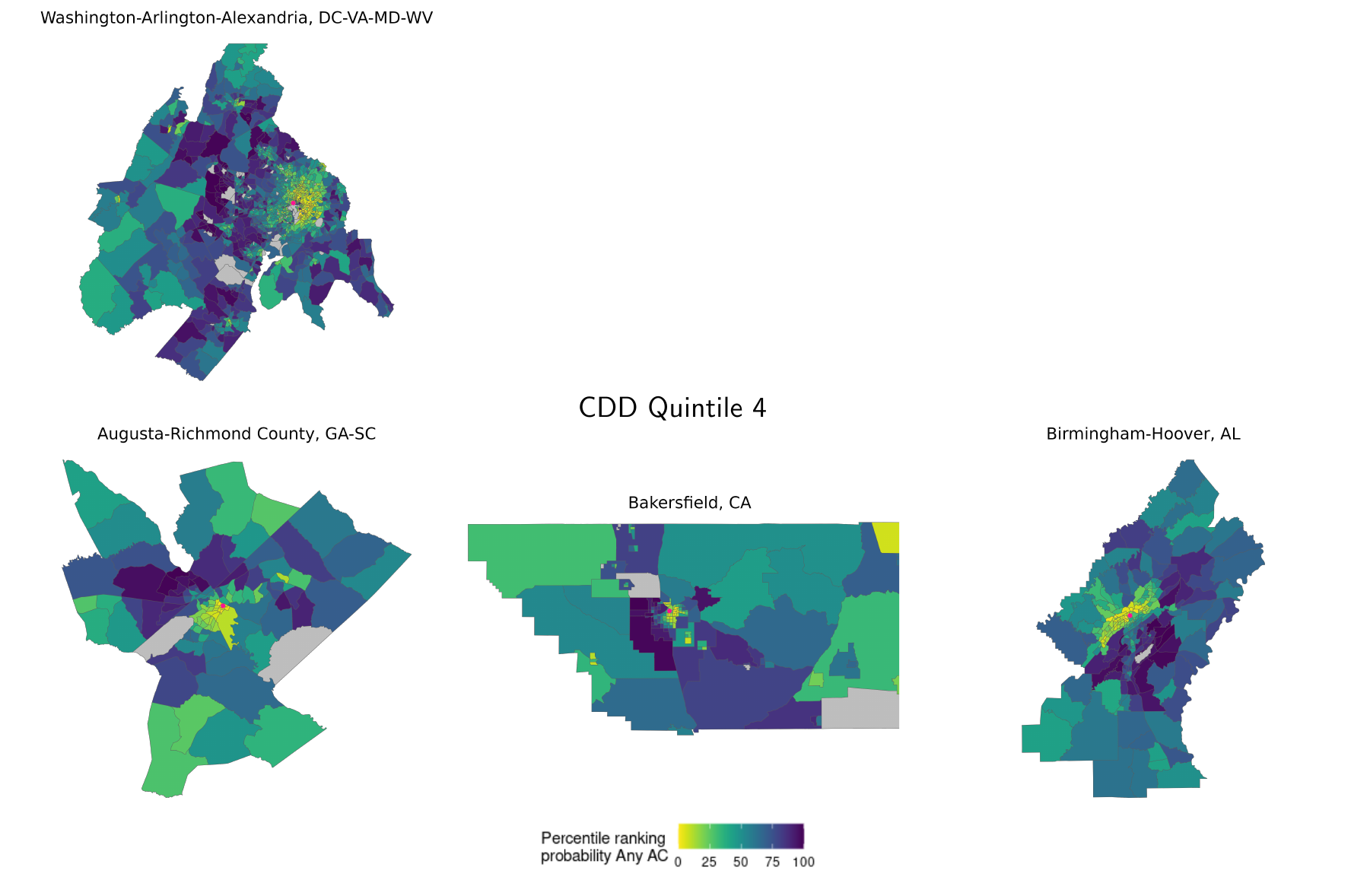

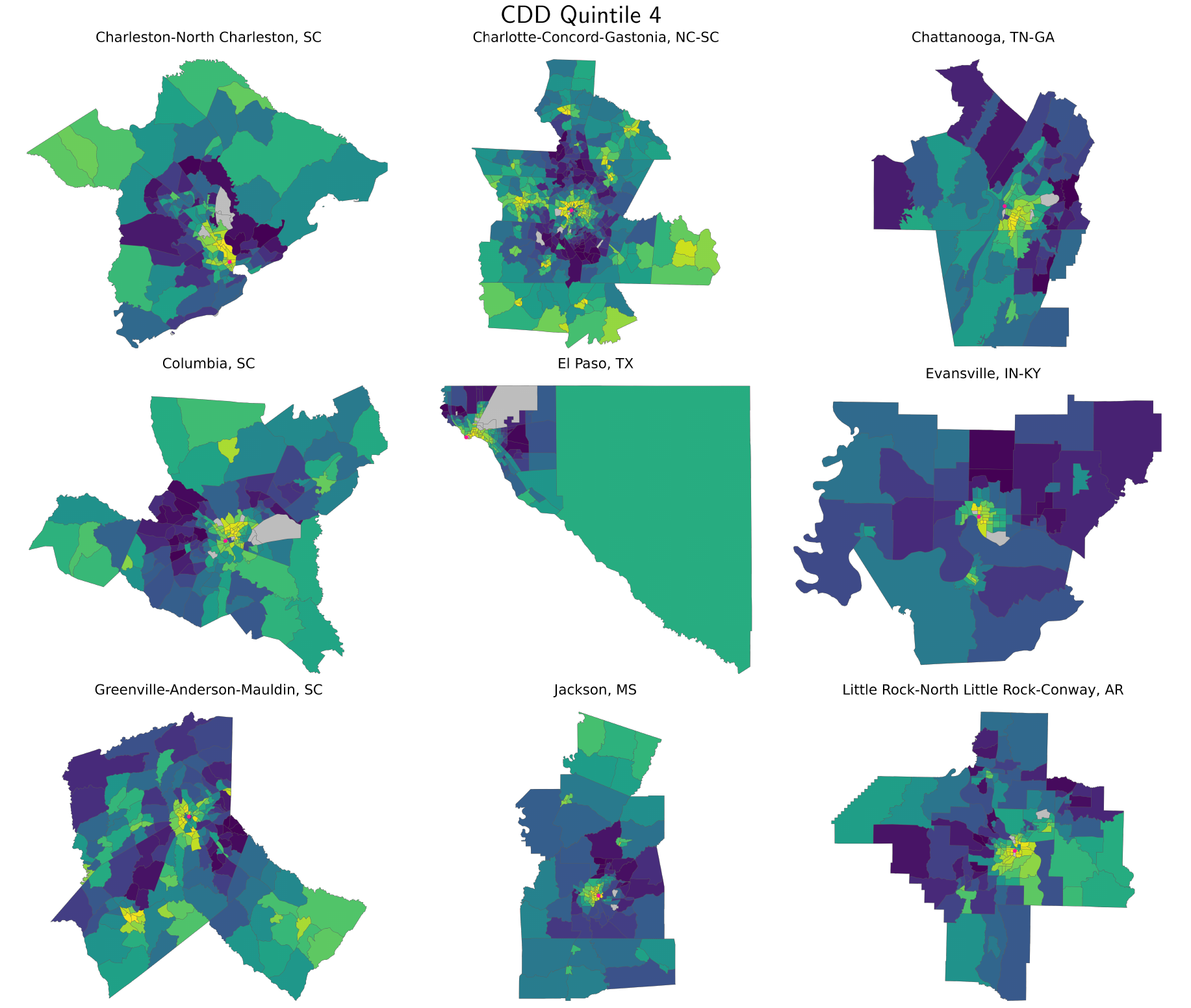

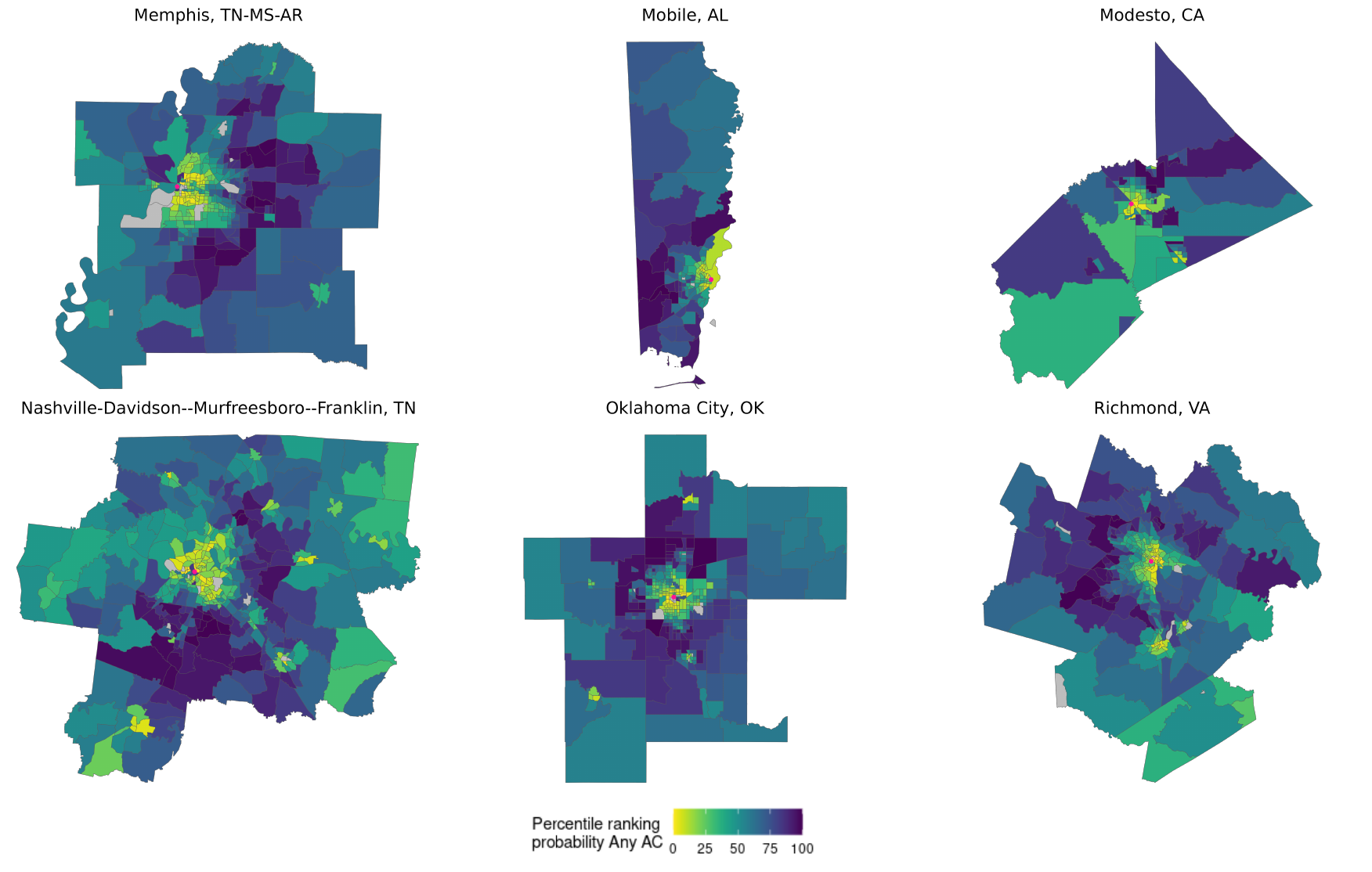

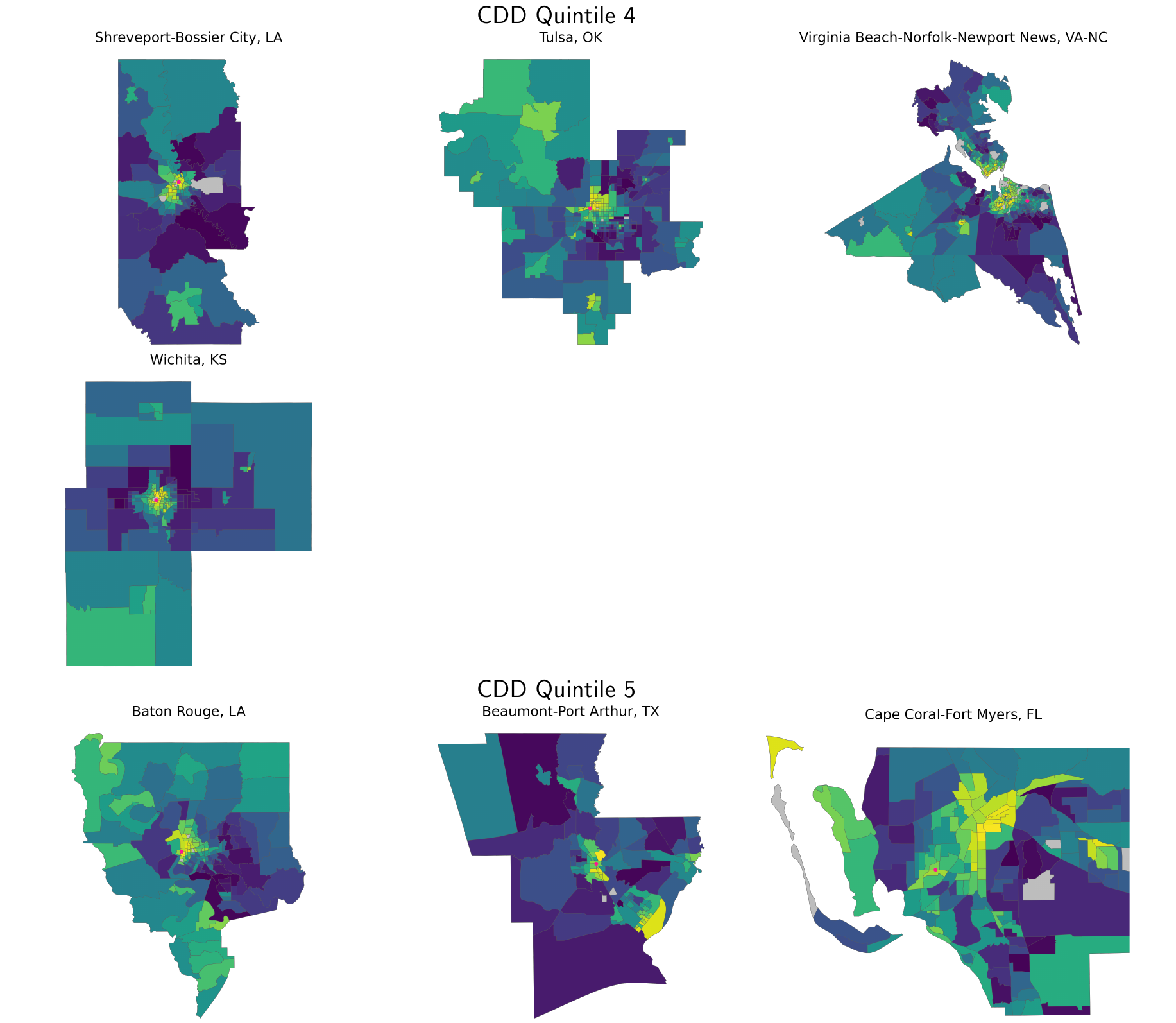

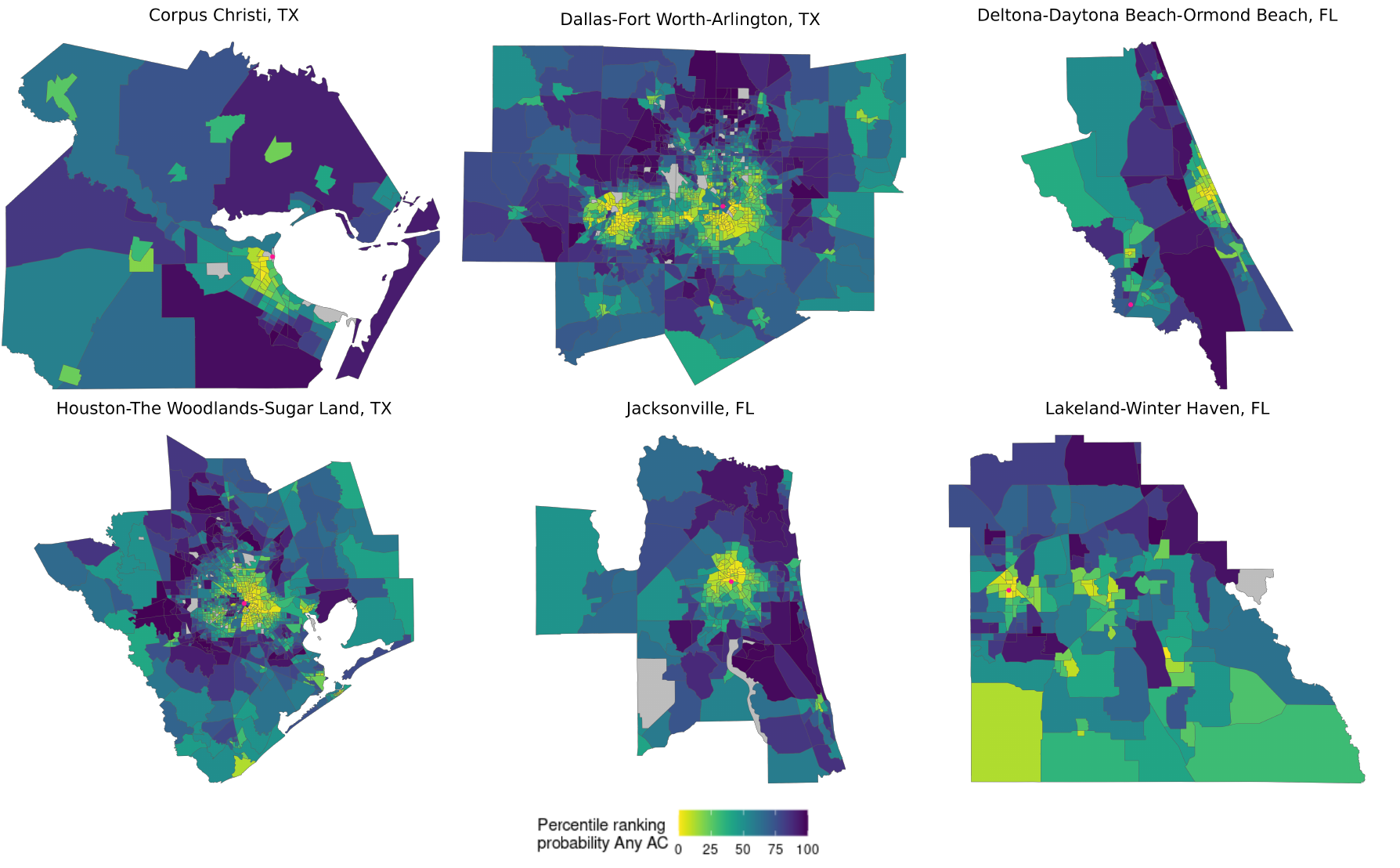

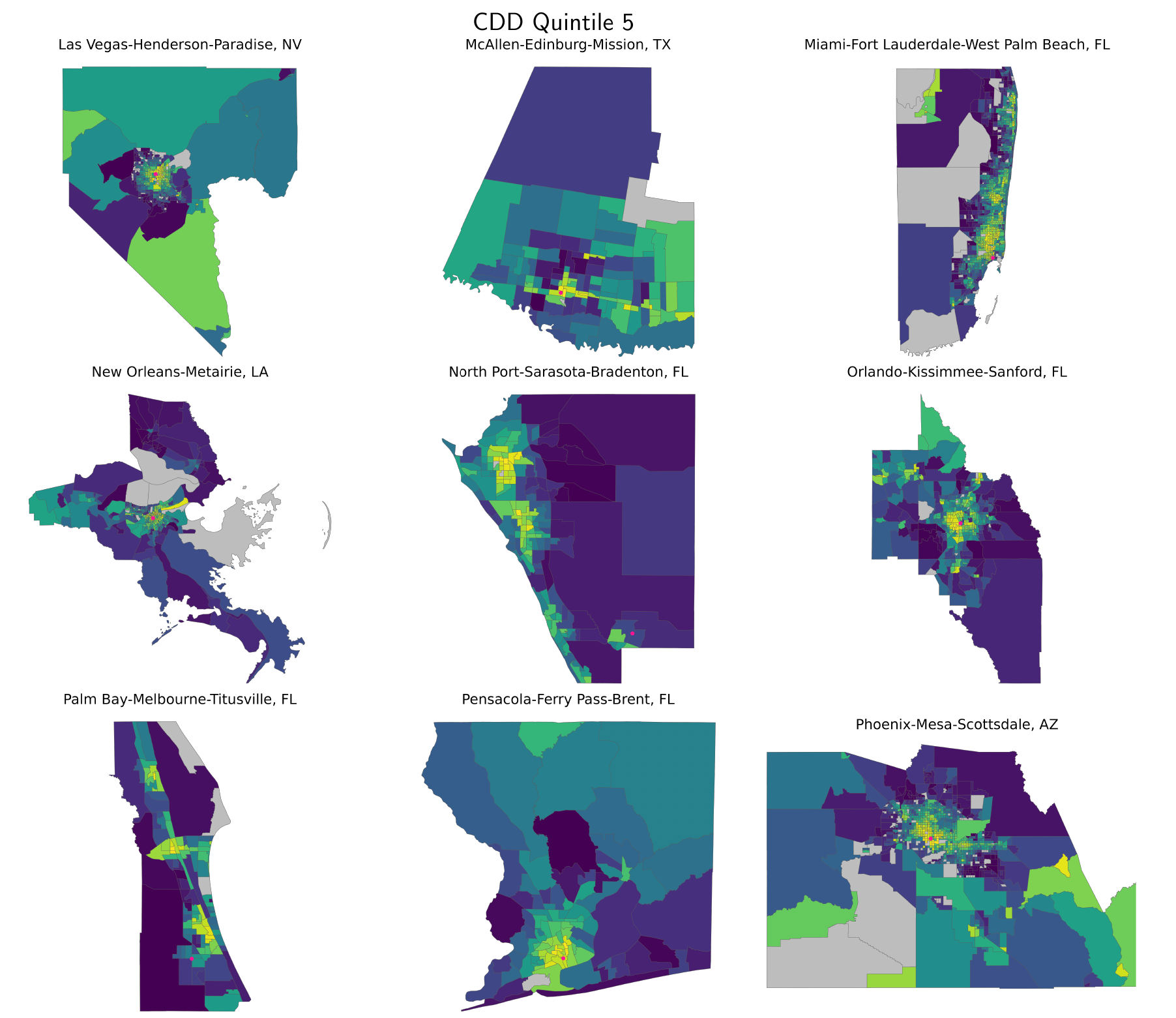

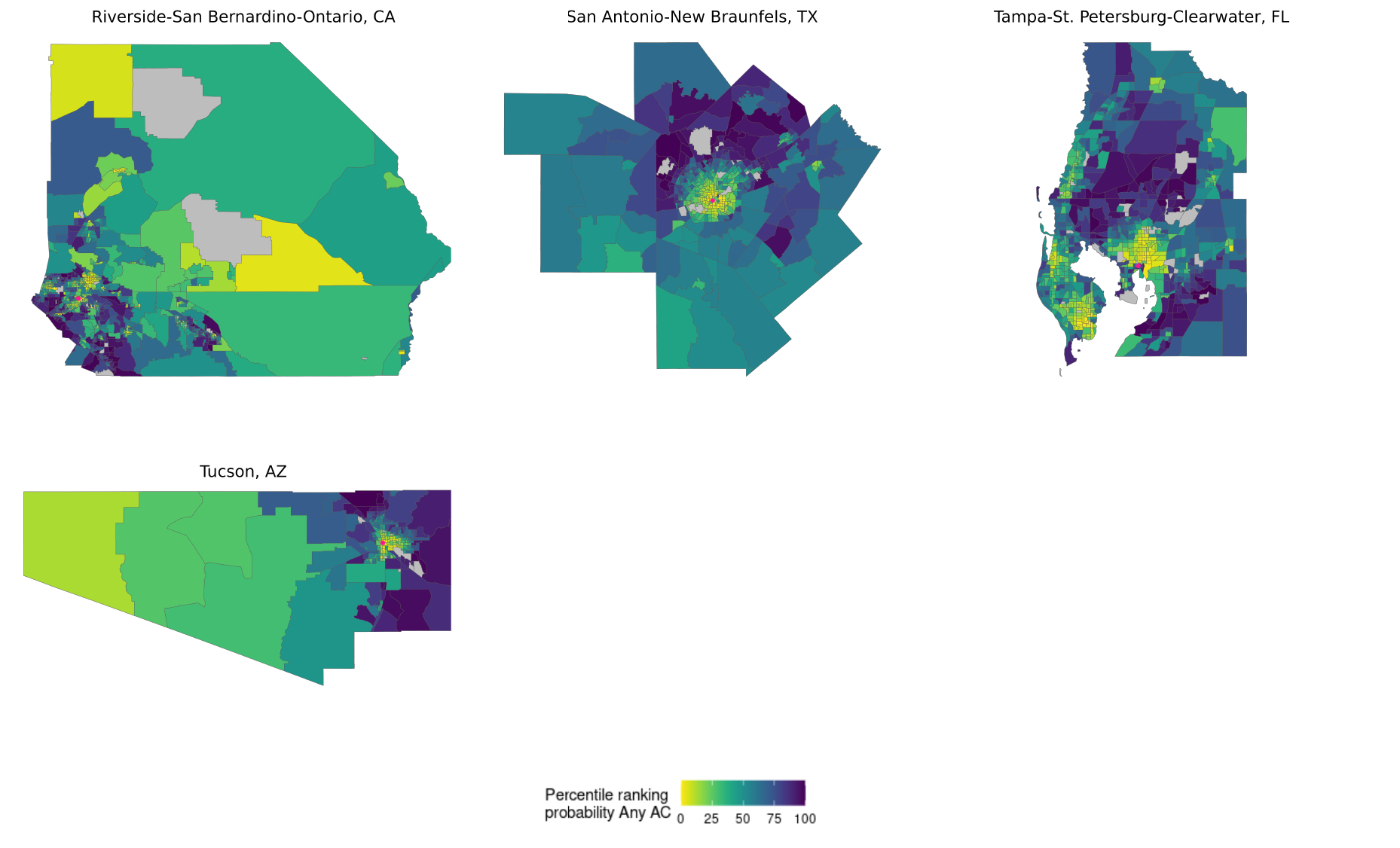


**Figure S4** Intra-urban variation in summer daytime UHI intensity (data extracted from Chakraborty et al, 2020) in 115 metropolitan areas. Black dots indicate the downtown or financial center of the metro area.


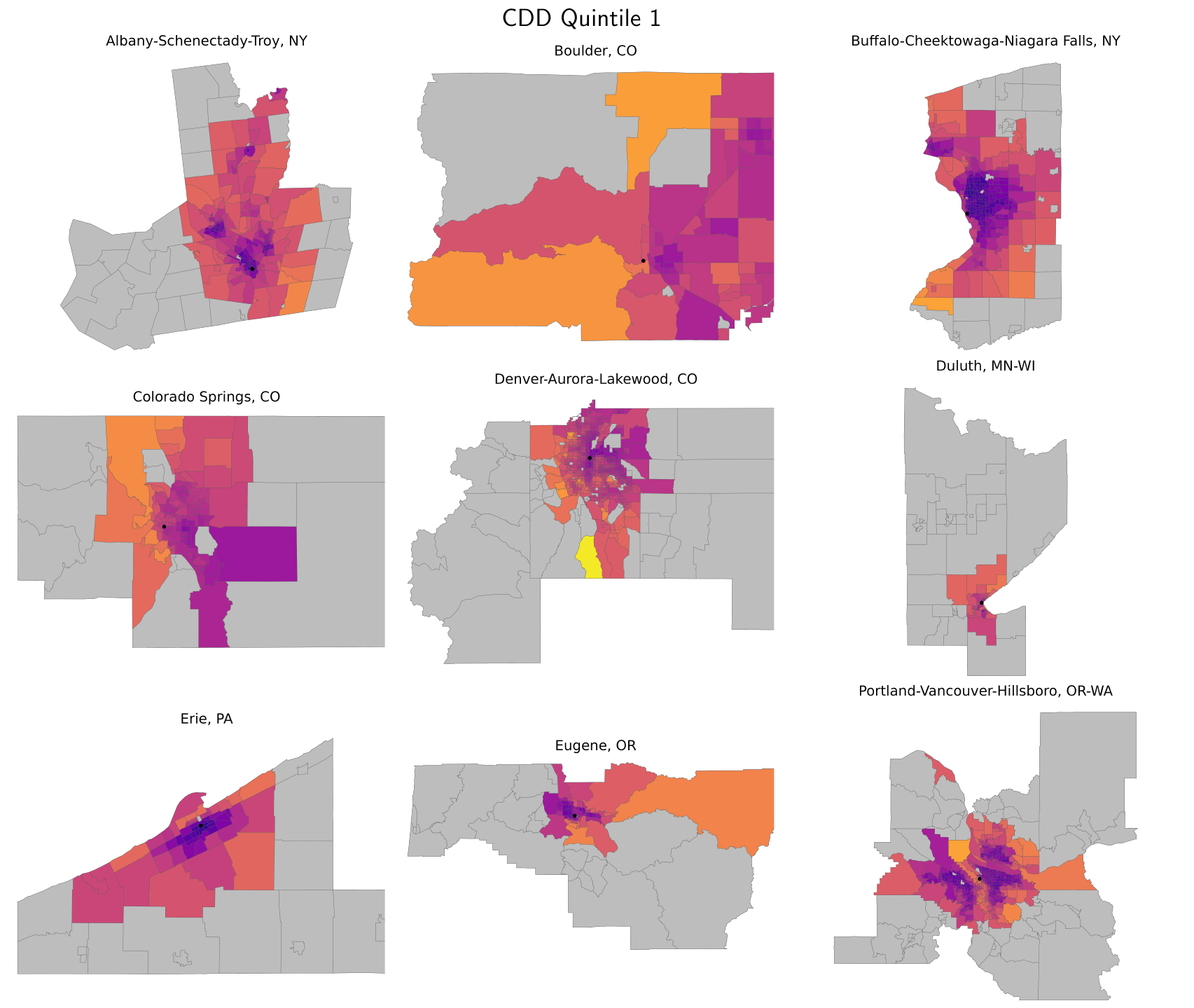

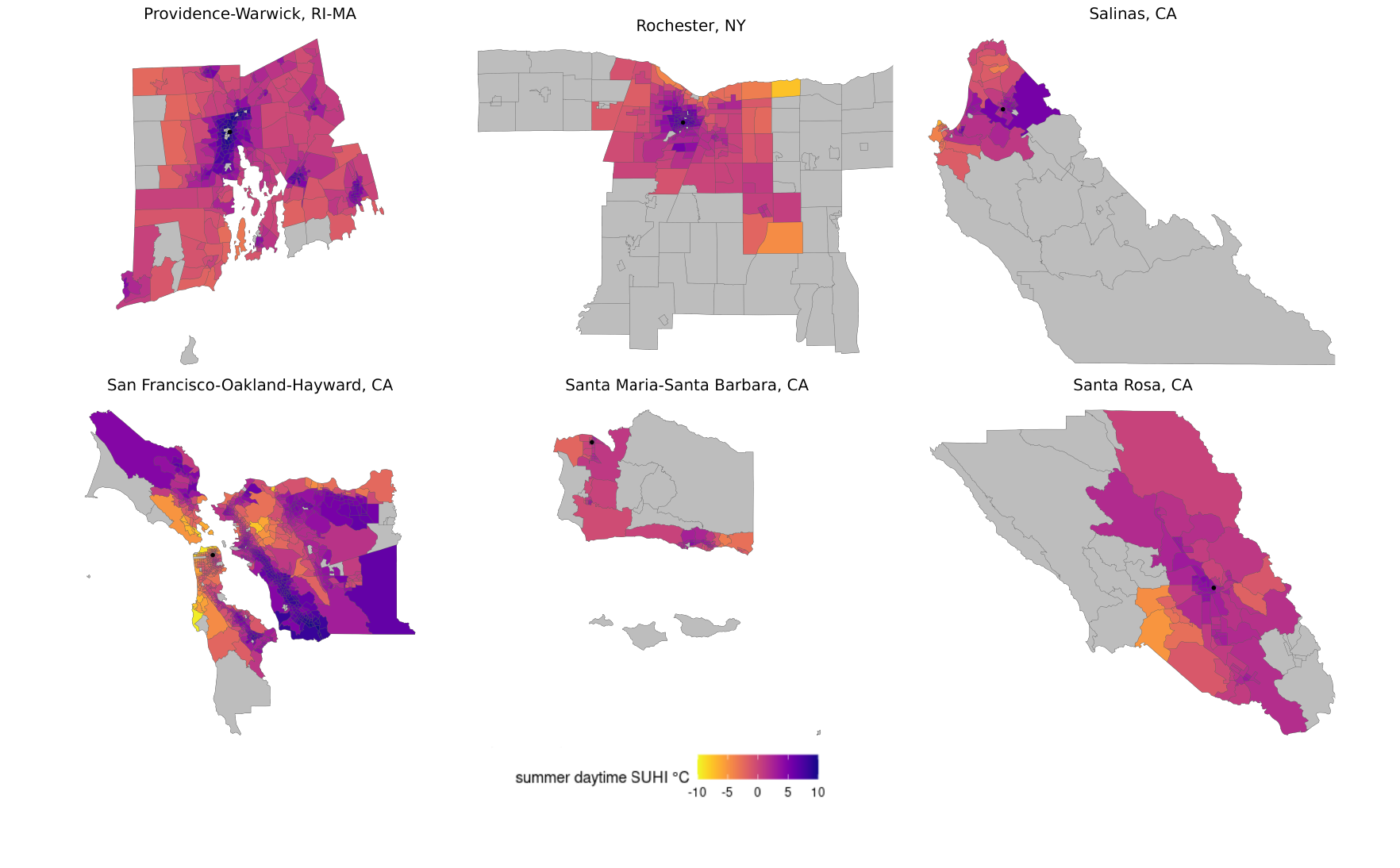

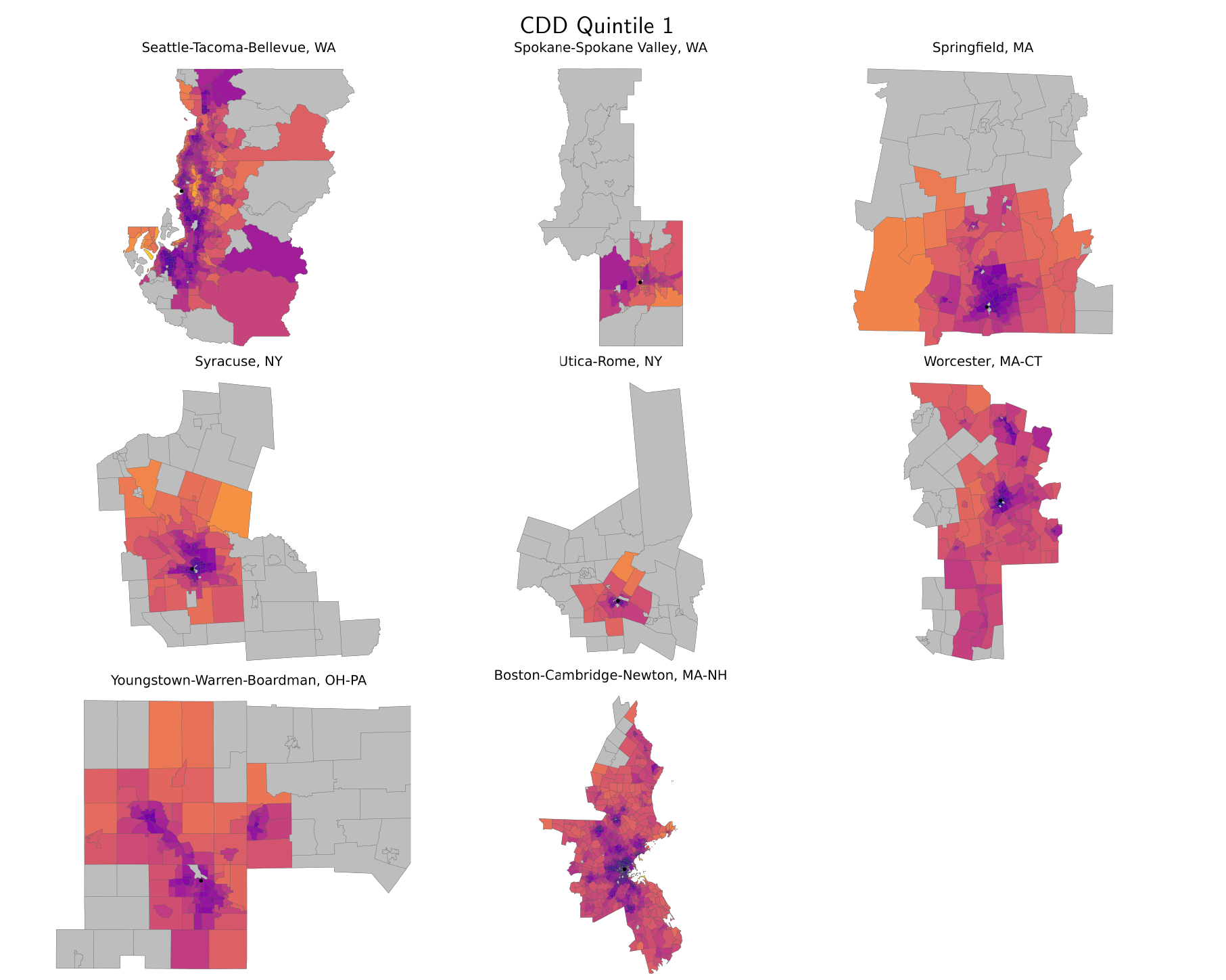

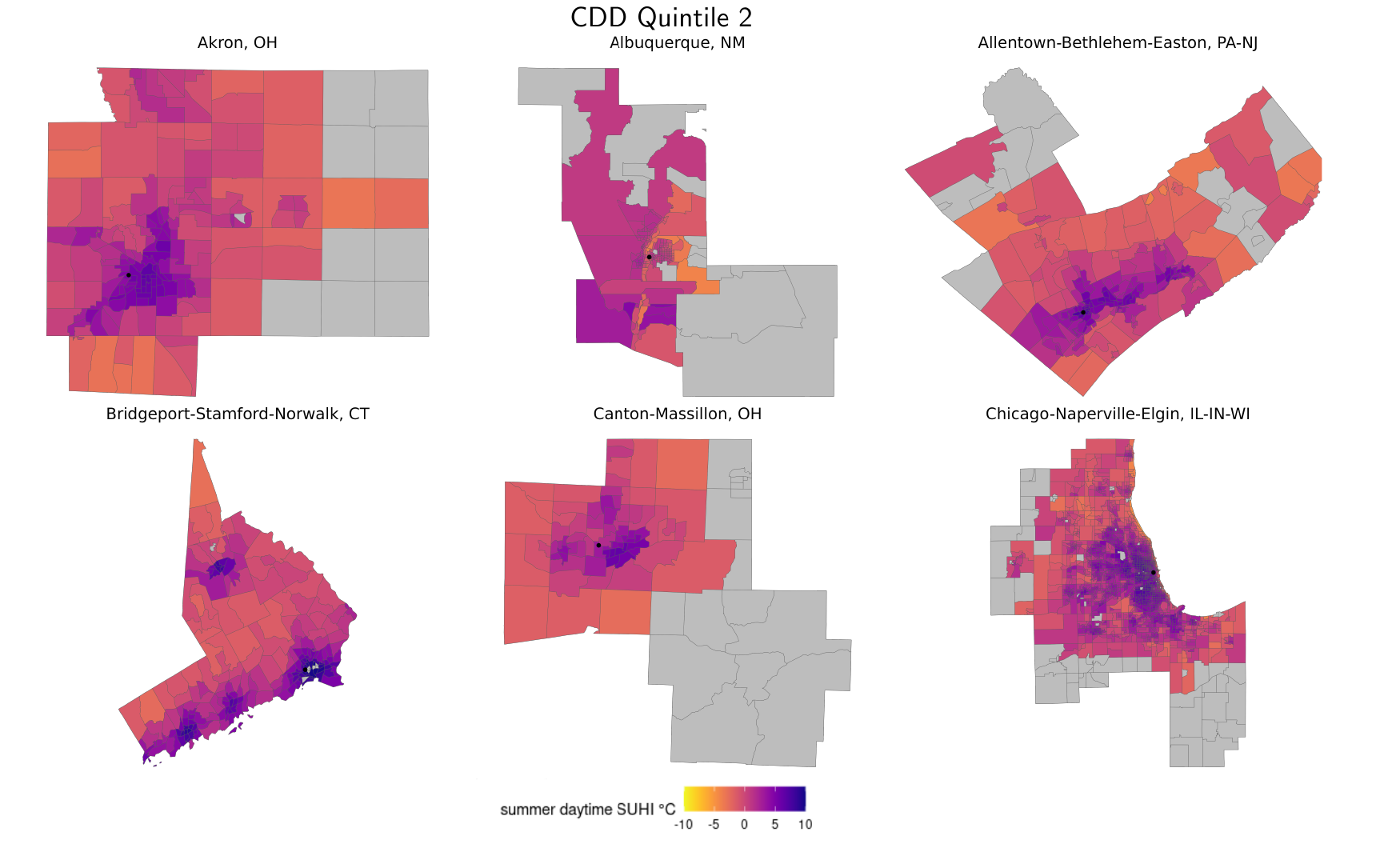

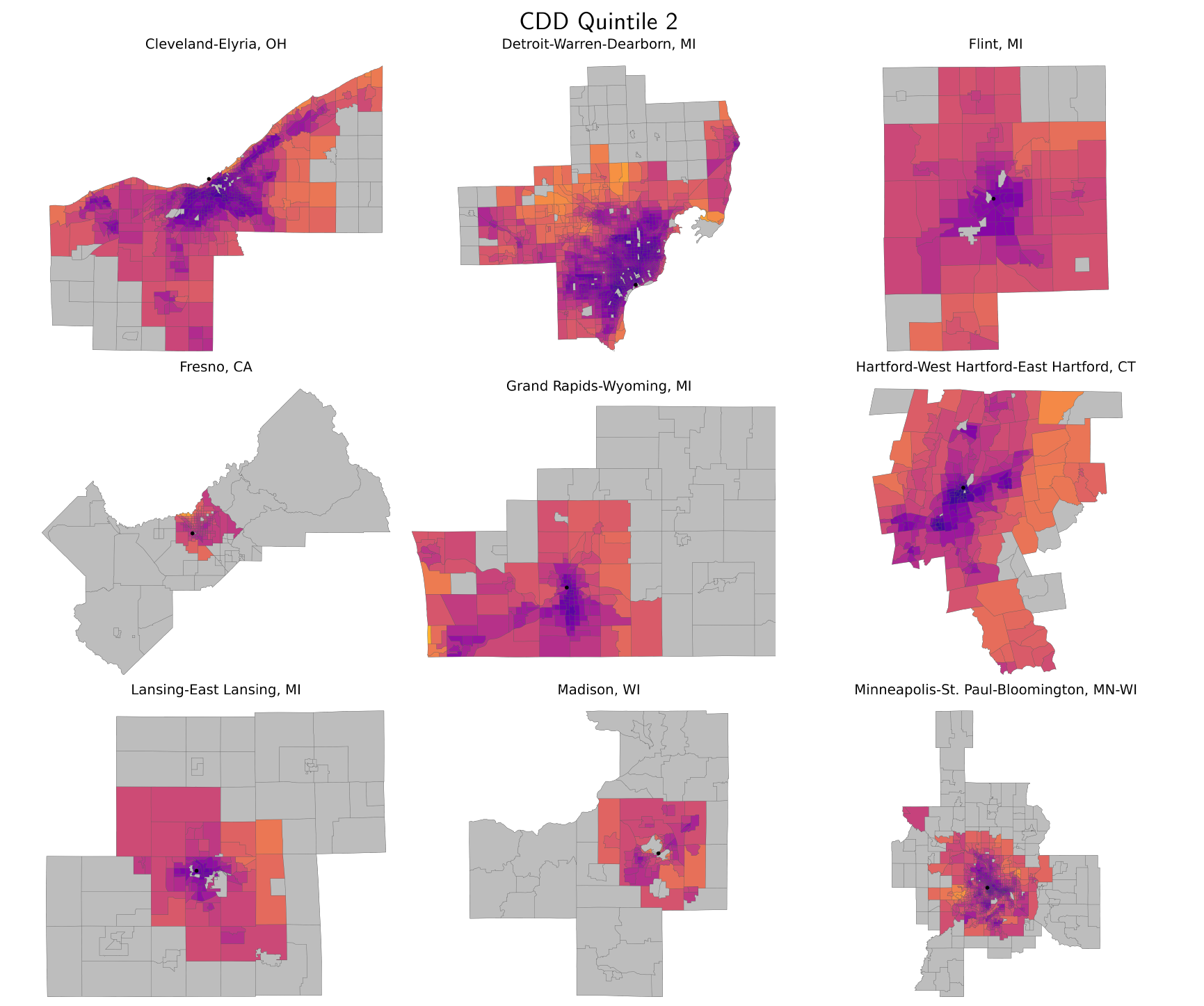

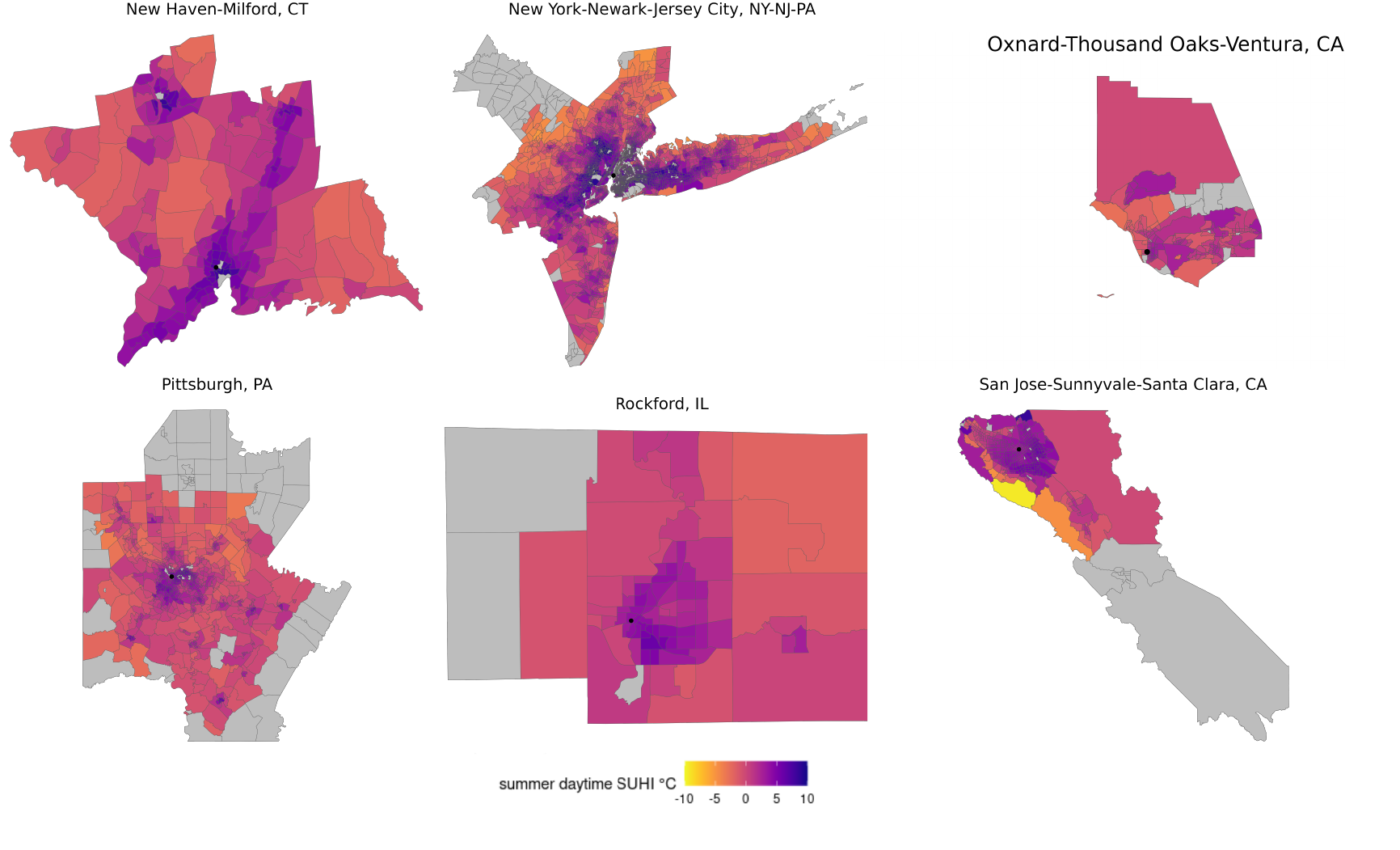

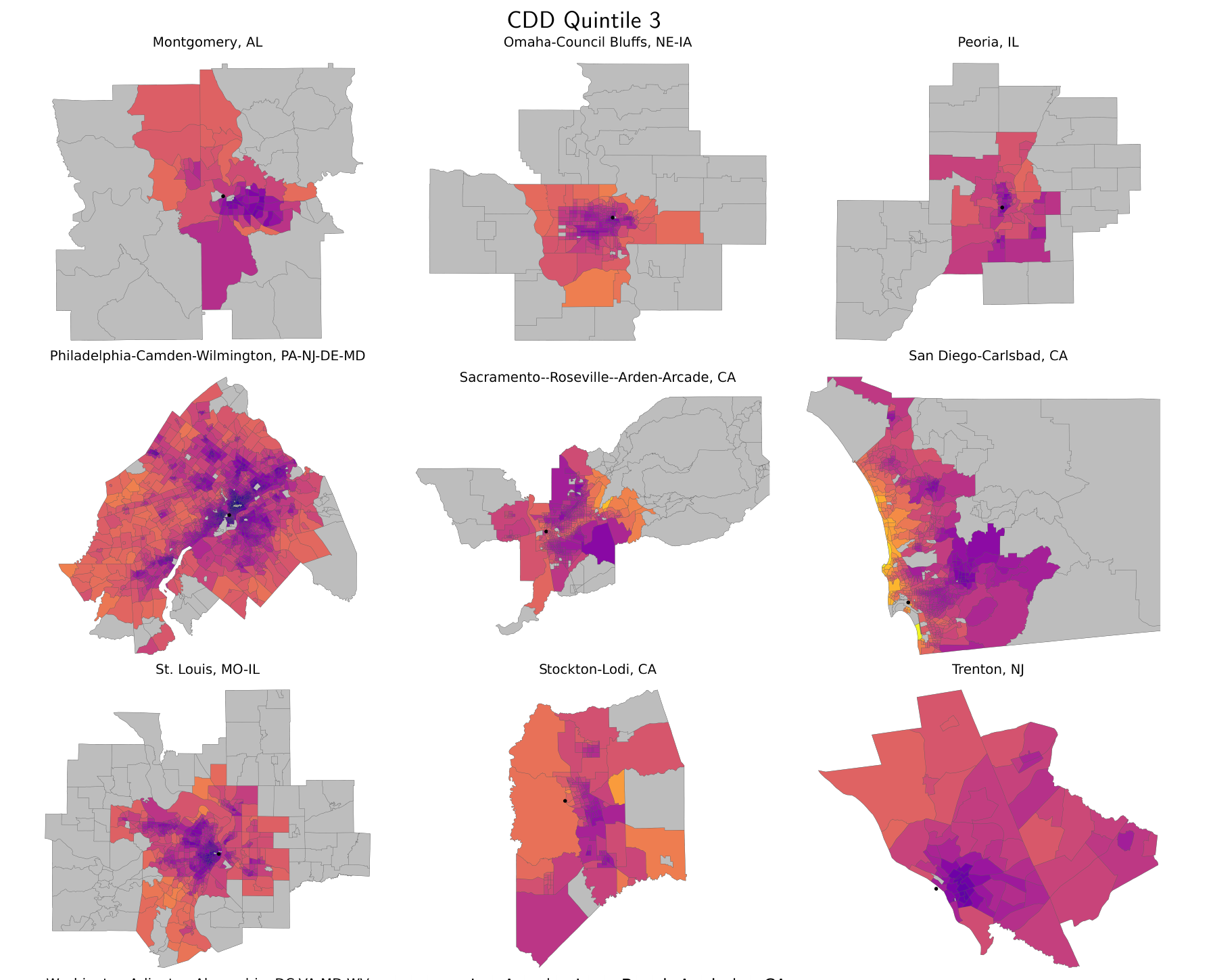

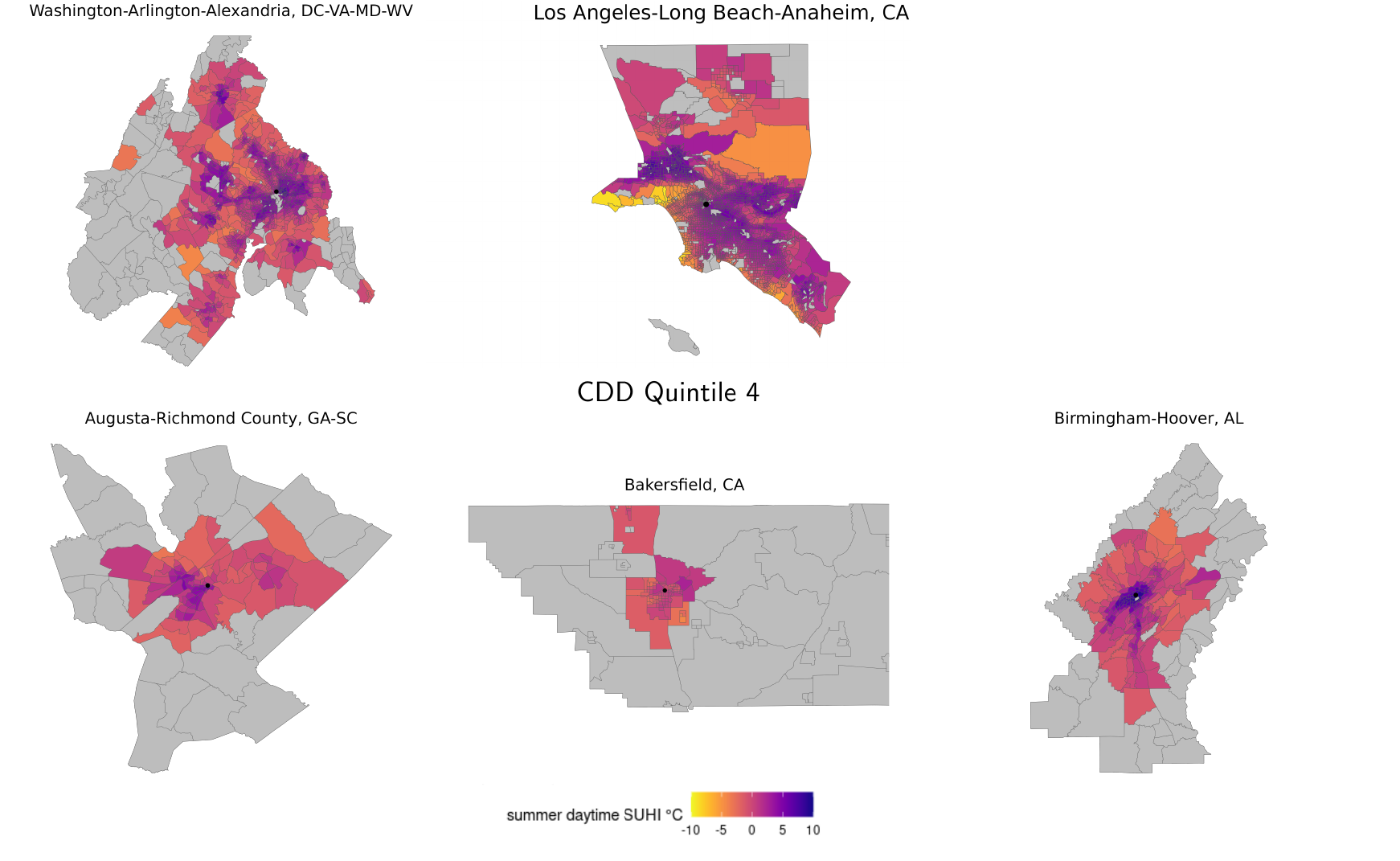

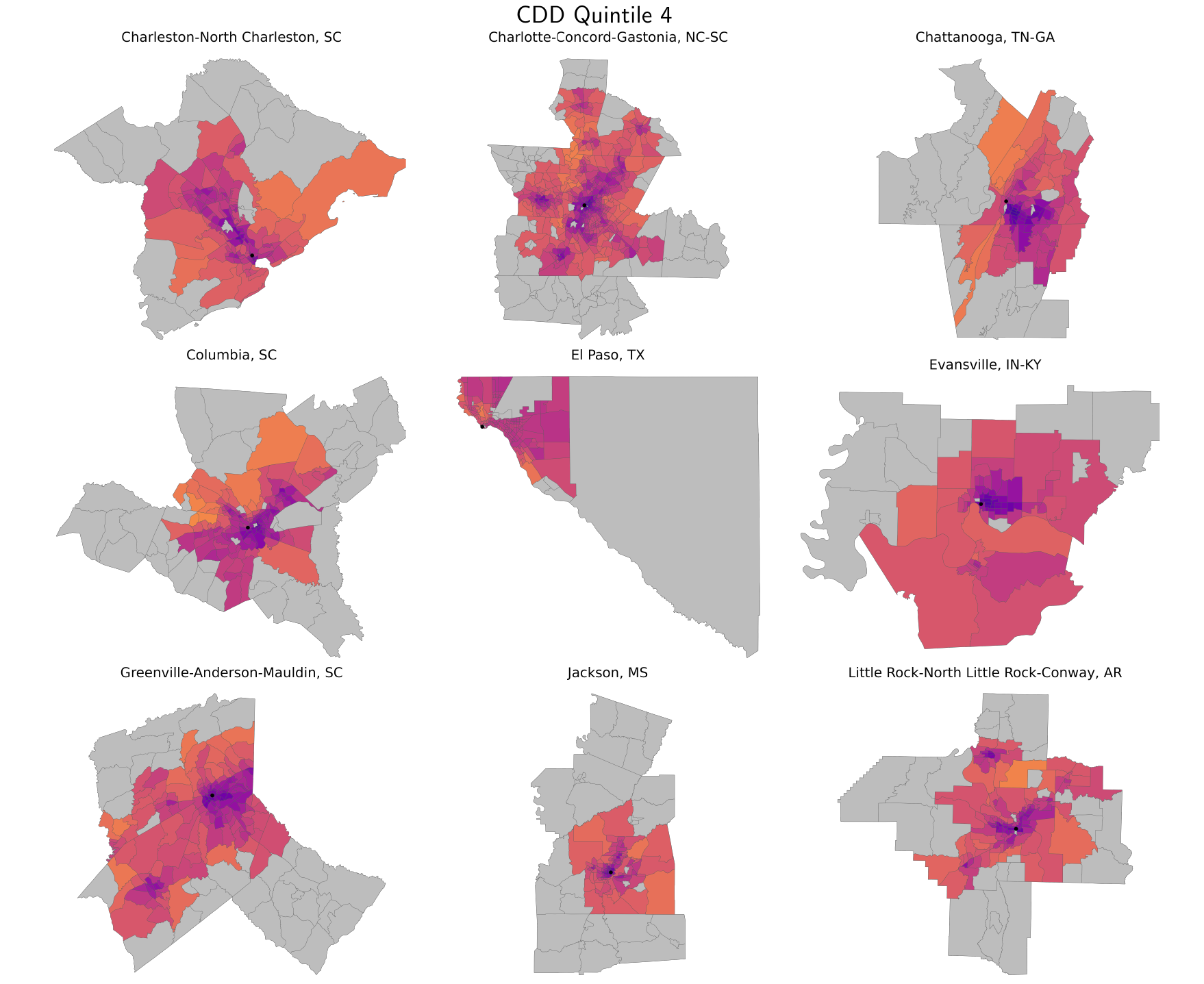

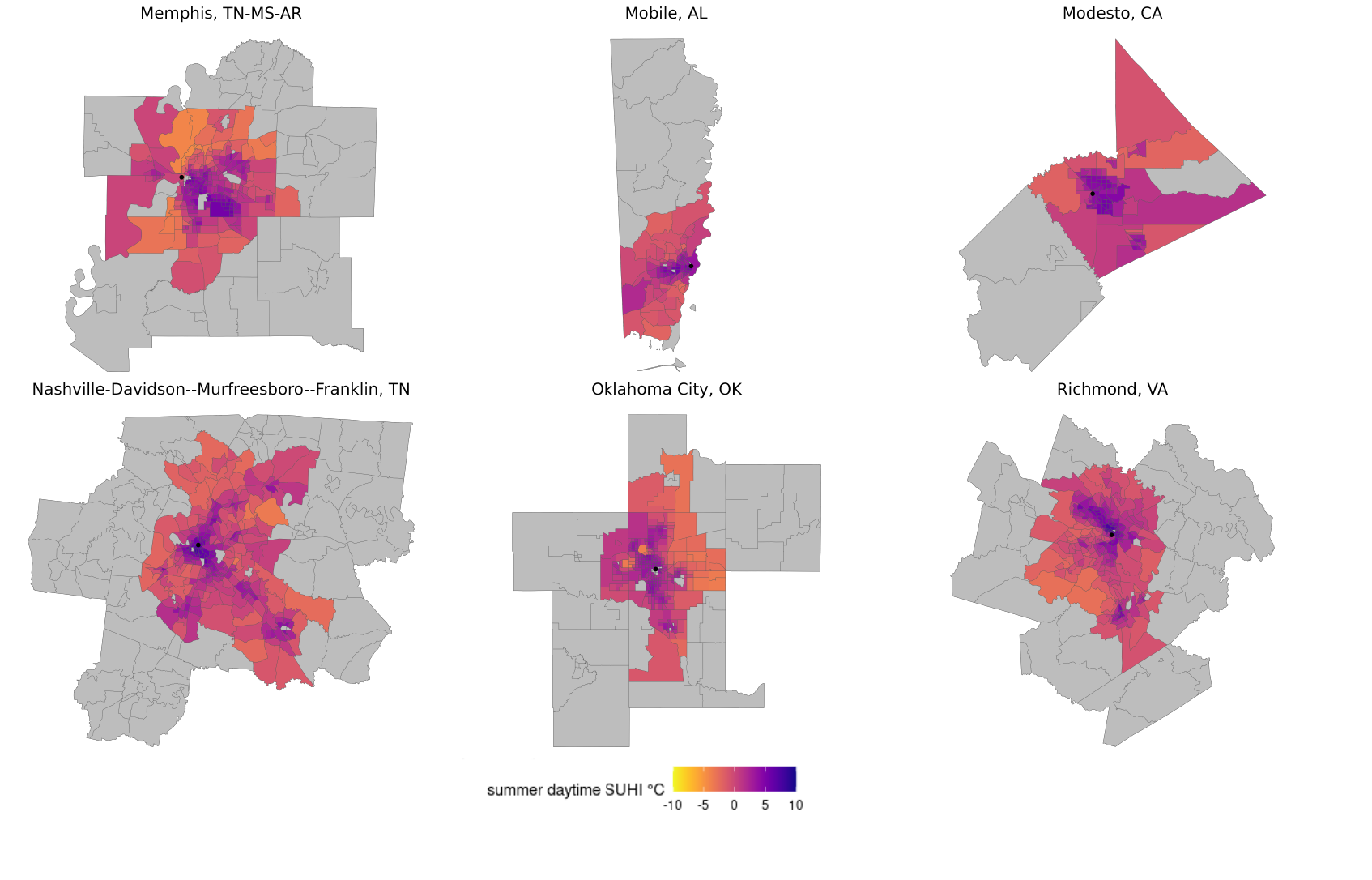

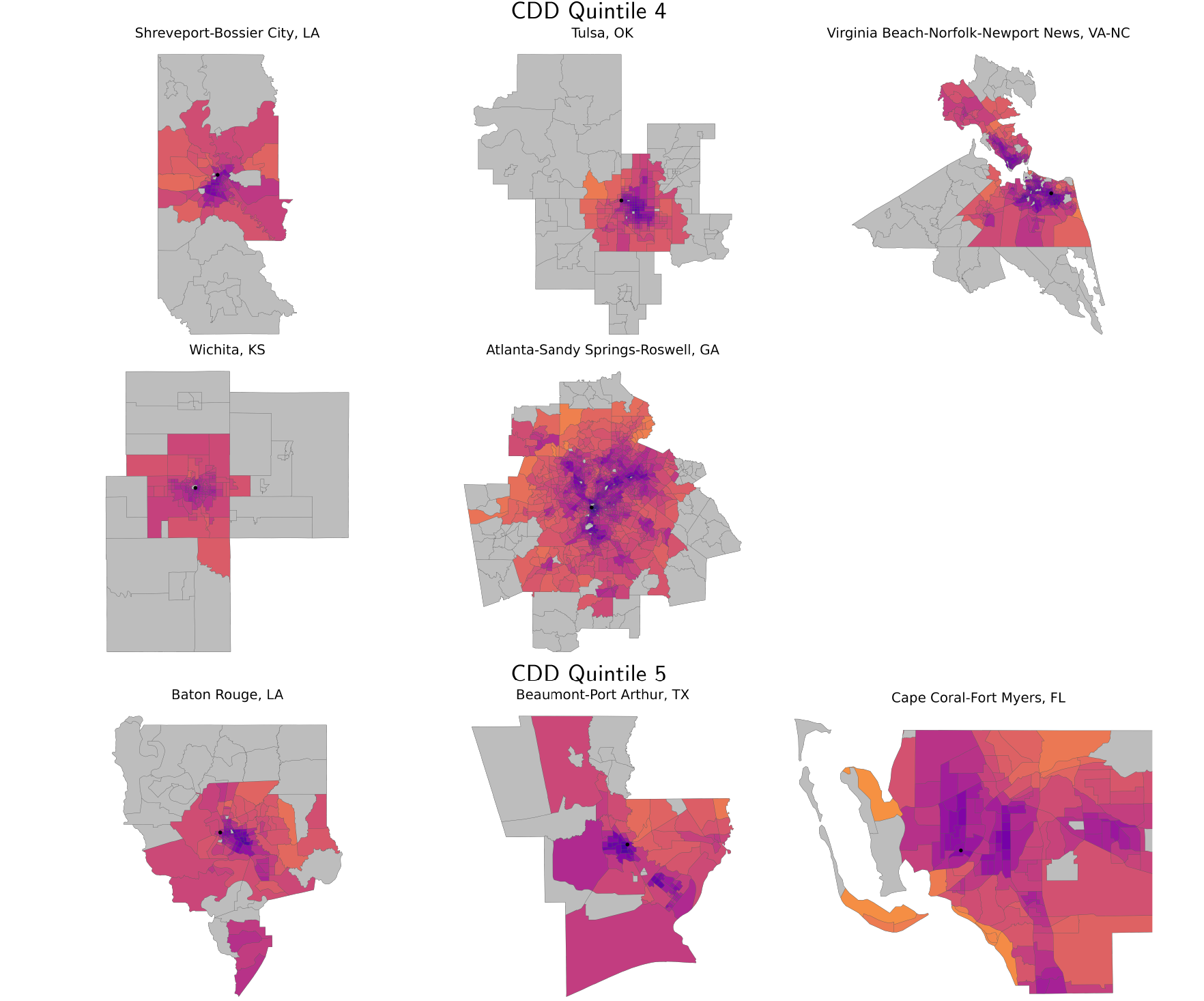

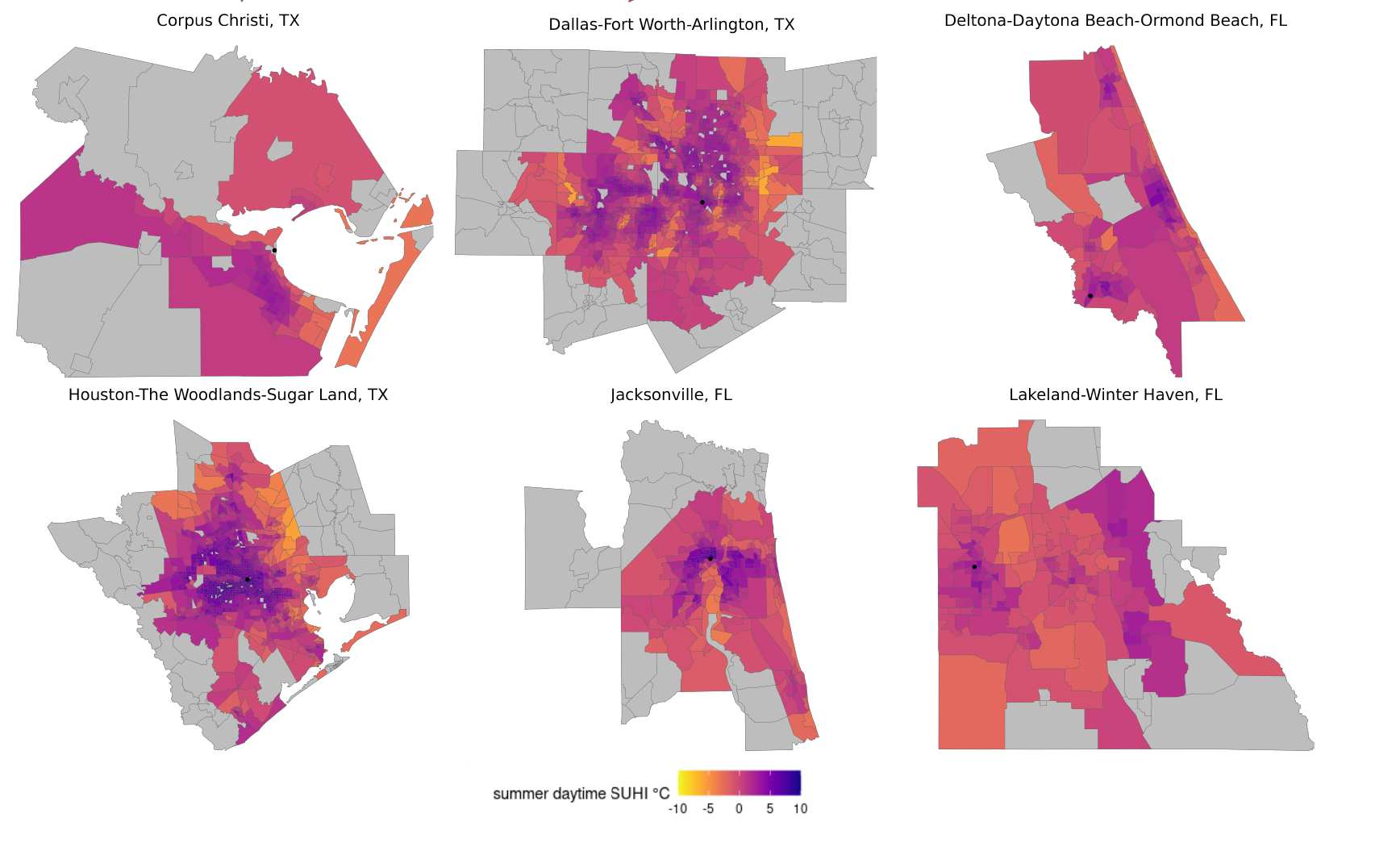

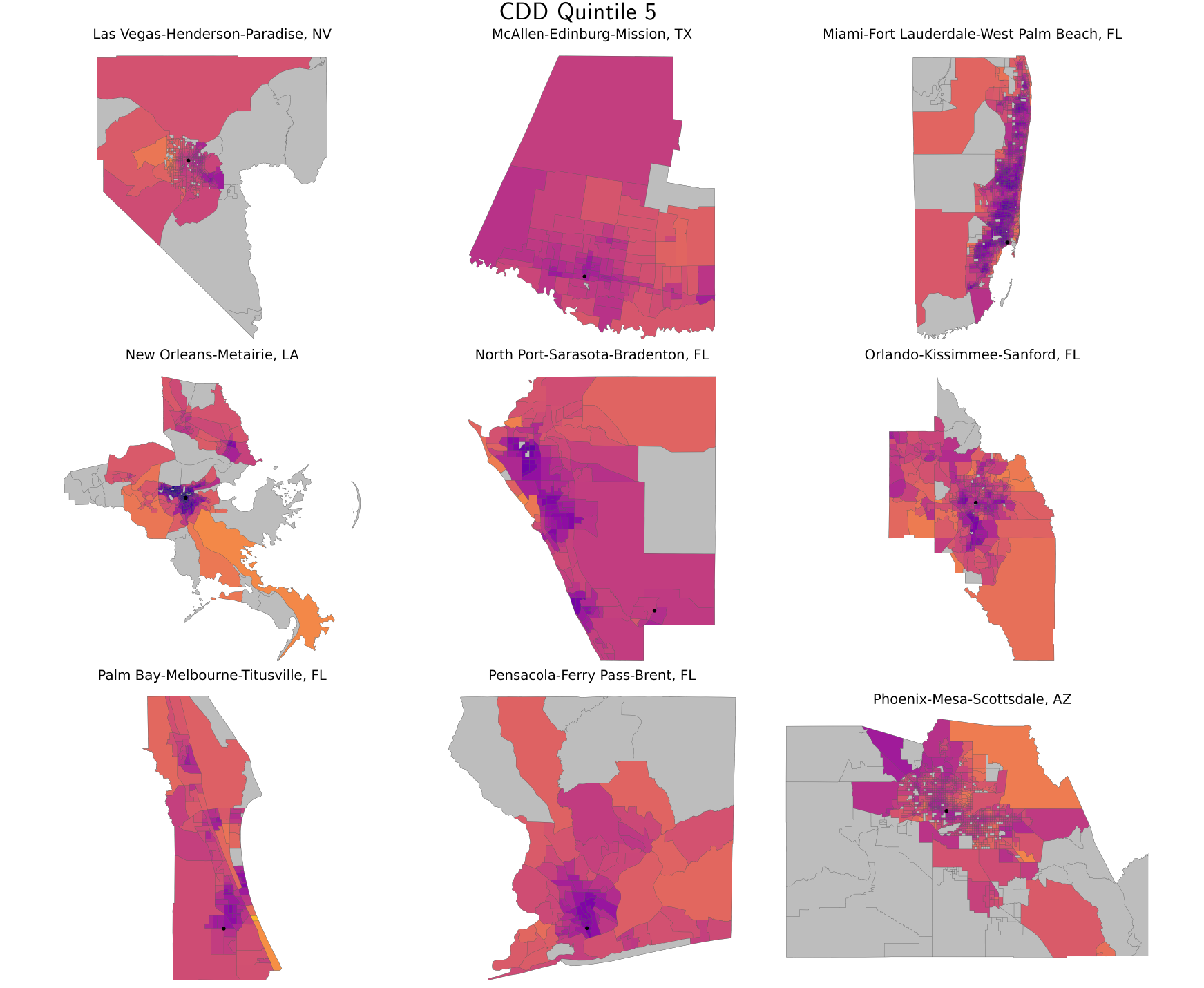

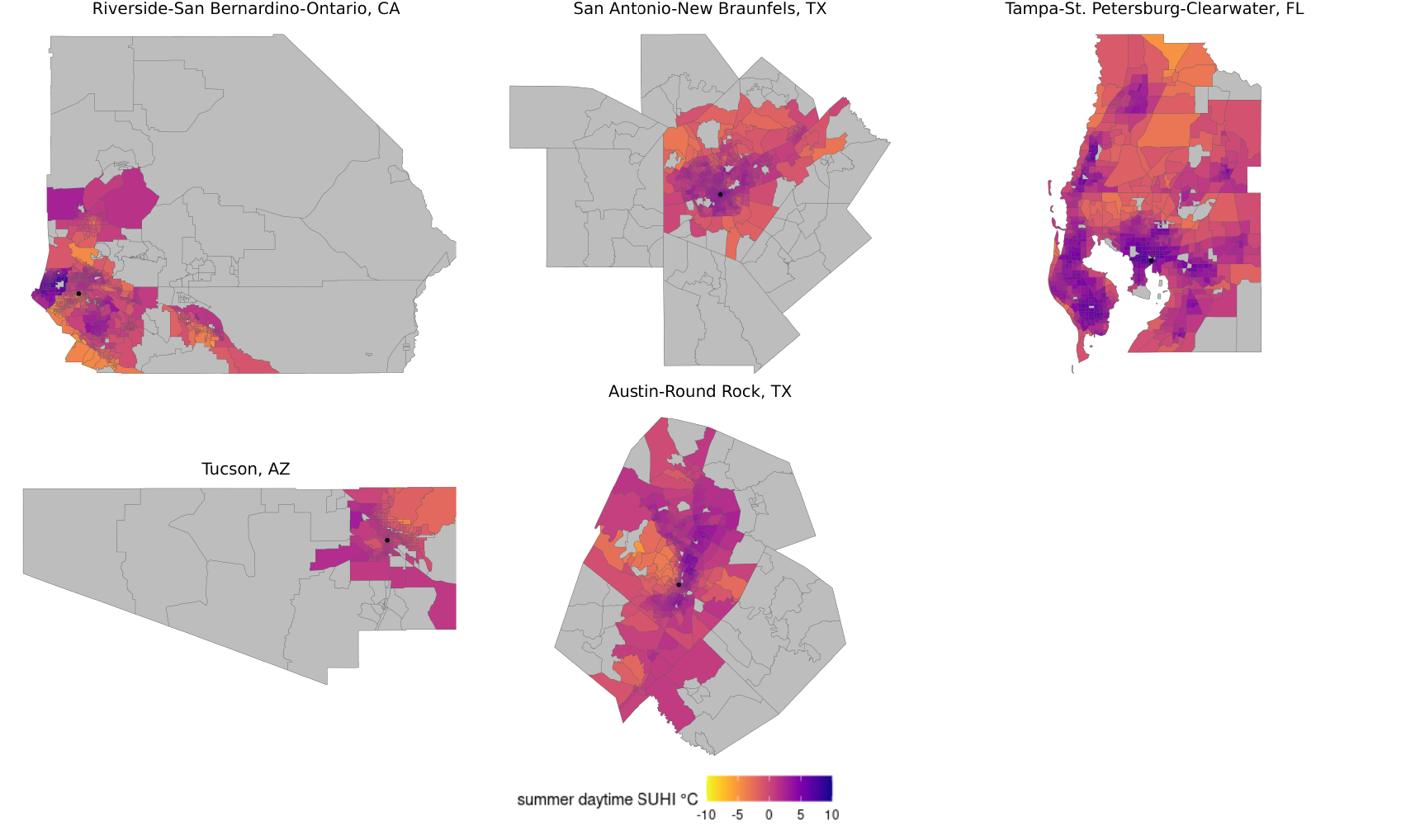


**Figure S5** Metropolitan area-level predicted probabilities of residential (any) AC compared to metropolitan area averages of (central) AC from Sera et al (2020) Supplementary eTable 1(d). Black lines denote 1:1 agreement.

**
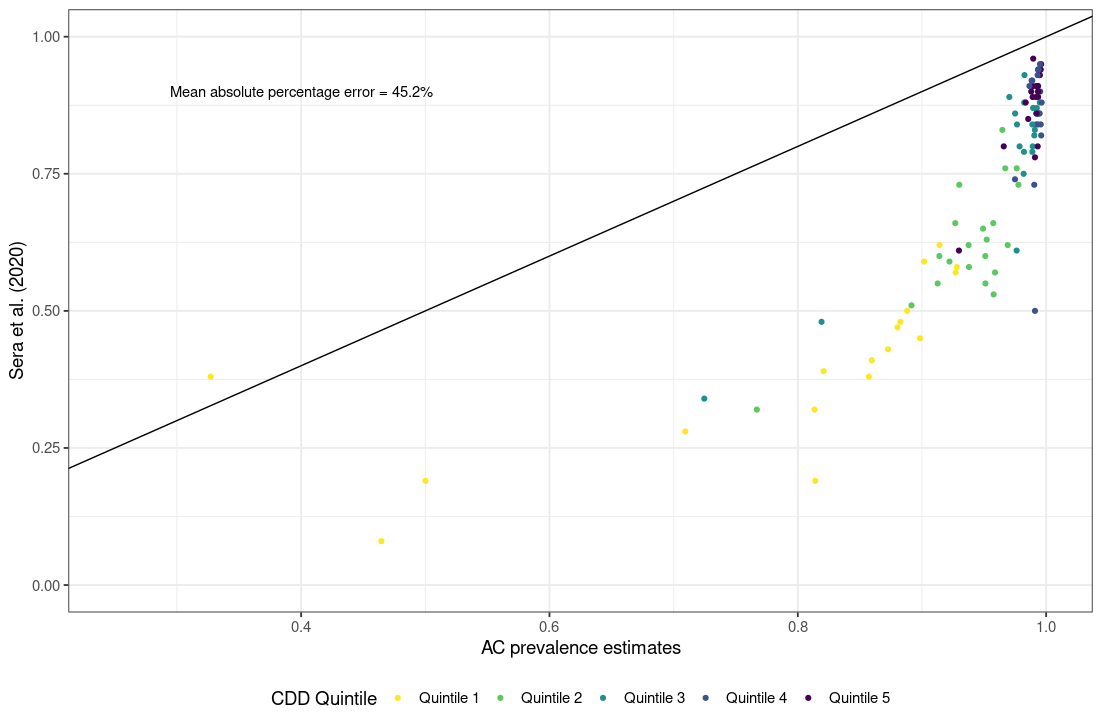
**


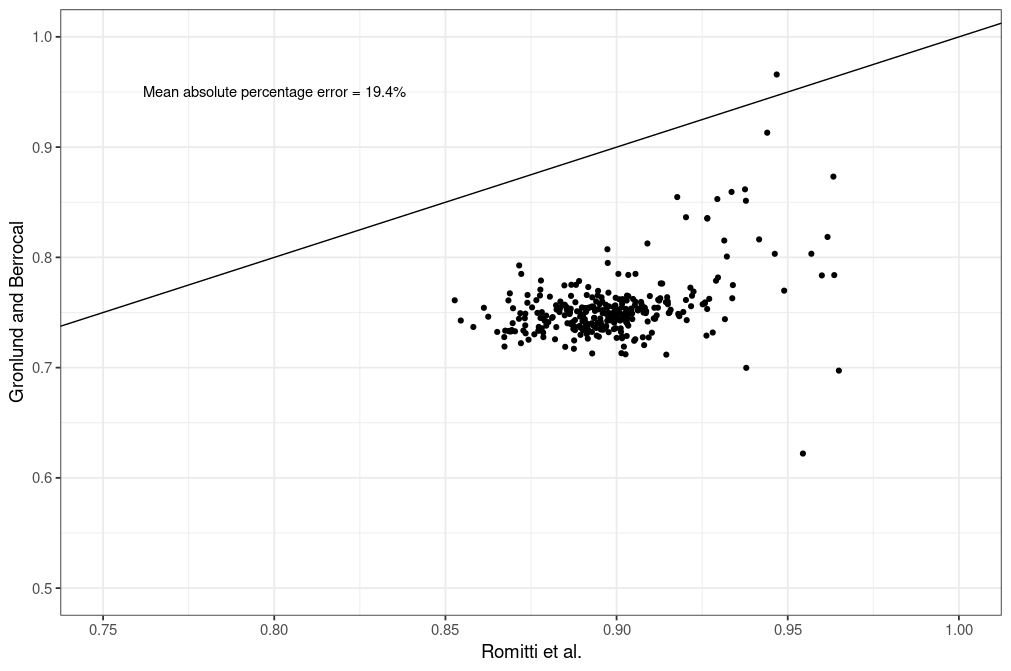
**Figure S6** Detroit census tract-level probabilities of residential (any) AC compared to tract level probabilities for Detroit in Gronlund and Berrocal (2020). Black line denotes 1:1 agreement between probabilities.


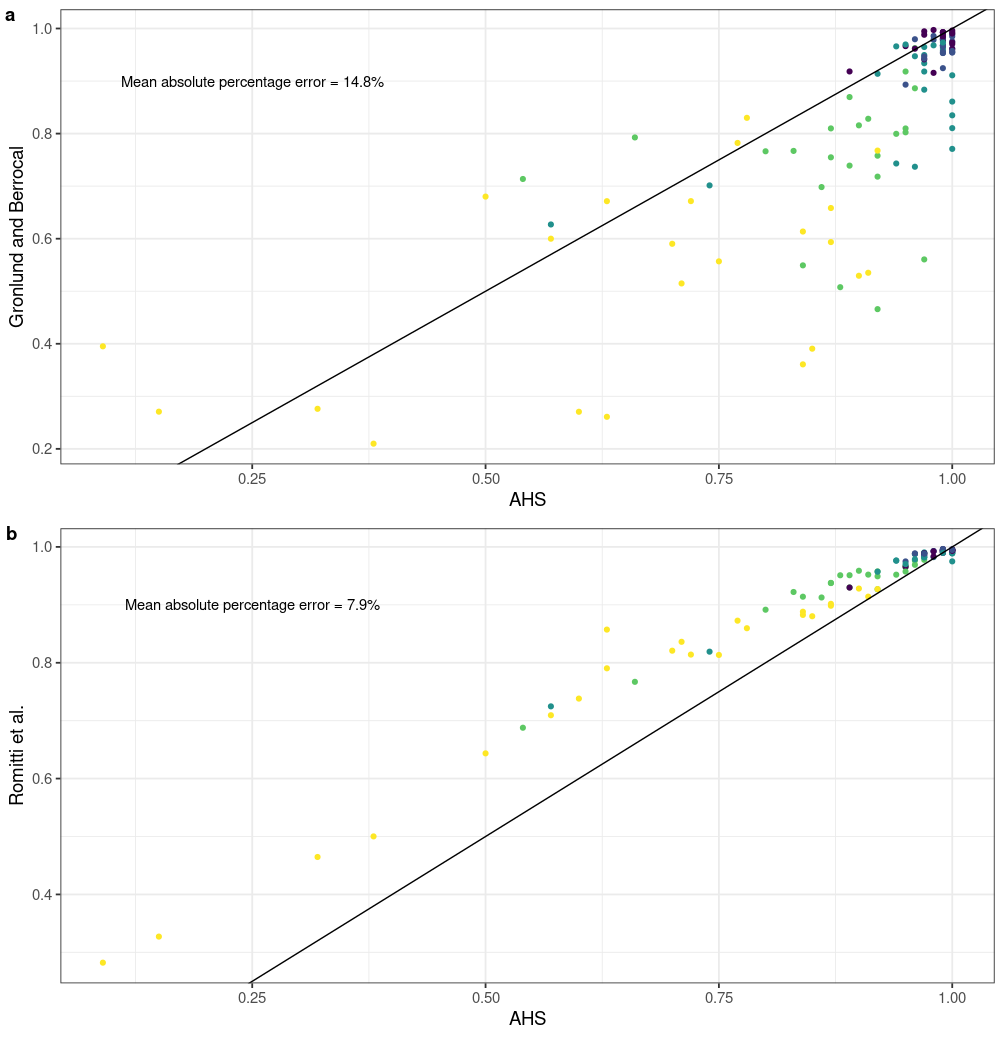
**Figure S7** (**a**) Population-weighted metropolitan area-level probabilities of residential (central) AC prevalence in Gronlund and Berrocal (2020) for the 115 cities in our sample compared to weighted (any) AC prevalence from AHS. (**b**) Our population-weighted metropolitan area-level probabilities of any AC compared to AHS. Black line denotes 1:1 agreement.

**Table S3**Metropolitan area climatic annual average cooling degree days (CDDs),computed as the area-weighted average of each county’s 30-year (1981–2010) CDDs for each metropolitan area, alongside the mean and standard deviation (SD) for predicted probabilities of tract residential (any) for each metropolitan area for our primary empirical specifications (1) and all alternate specifications (A1-A3).

| **Metropolitan area** | **CDDs** | **Mean (1)** | **SD (1)** | **Mean (A1)** | **SD (A1)** | **Mean (A2)** | **SD (A2)** | **Mean (A3)** | **SD (A3)** |
| --- | --- | --- | --- | --- | --- | --- | --- | --- | --- |
| Akron, OH | 595 | 0.90 | 0.05 | 0.90 | 0.05 | 0.91 | 0.05 | 0.91 | 0.04 |
| Albany-Schenectady-Troy, NY | 358 | 0.89 | 0.05 | 0.92 | 0.05 | 0.90 | 0.05 | 0.93 | 0.04 |
| Albuquerque, NM | 527 | 0.97 | 0.01 | 0.97 | 0.01 | 0.97 | 0.01 | 0.98 | 0.01 |
| Allentown-Bethlehem-Easton, PA-NJ | 540 | 0.98 | 0.01 | 0.98 | 0.01 | 0.98 | 0.01 | 0.99 | 0.01 |
| Atlanta-Sandy Springs-Roswell, GA | 1570 | 0.99 | 0.00 | 0.99 | 0.00 | 0.99 | 0.00 | 0.99 | 0.00 |
| Atlantic City-Hammonton, NJ | 909 | 0.97 | 0.01 | 0.96 | 0.02 | 0.98 | 0.01 | 0.98 | 0.01 |
| Augusta-Richmond County, GA-SC | 1980 | 0.99 | 0.00 | 0.99 | 0.01 | 0.99 | 0.00 | 0.99 | 0.01 |
| Austin-Round Rock, TX | 2747 | 1.00 | 0.00 | 1.00 | 0.00 | 1.00 | 0.00 | 1.00 | 0.00 |
| Bakersfield, CA | 1687 | 0.97 | 0.01 | 0.96 | 0.02 | 0.98 | 0.01 | 0.98 | 0.01 |
| Baltimore-Columbia-Towson, MD | 1045 | 0.99 | 0.01 | 0.99 | 0.01 | 0.99 | 0.01 | 0.99 | 0.01 |
| Baton Rouge, LA | 2554 | 0.99 | 0.00 | 1.00 | 0.00 | 0.99 | 0.00 | 0.99 | 0.00 |
| Beaumont-Port Arthur, TX | 2605 | 0.99 | 0.01 | 0.99 | 0.01 | 0.99 | 0.01 | 0.99 | 0.00 |
| Birmingham-Hoover, AL | 1817 | 0.99 | 0.01 | 0.99 | 0.01 | 0.99 | 0.01 | 0.99 | 0.01 |
| Boston-Cambridge-Newton, MA-NH | 464 | 0.93 | 0.03 | 0.92 | 0.04 | 0.92 | 0.04 | 0.94 | 0.03 |
| Boulder, CO | 146 | 0.86 | 0.05 | 0.87 | 0.05 | 0.86 | 0.06 | 0.88 | 0.05 |
| Bridgeport-Stamford-Norwalk, CT | 605 | 0.93 | 0.06 | 0.93 | 0.04 | 0.93 | 0.10 | 0.93 | 0.05 |
| Buffalo-Cheektowaga-Niagara Falls, NY | 438 | 0.80 | 0.08 | 0.83 | 0.07 | 0.82 | 0.07 | 0.84 | 0.07 |
| Canton-Massillon, OH | 605 | 0.92 | 0.04 | 0.92 | 0.04 | 0.92 | 0.04 | 0.93 | 0.03 |
| Cape Coral-Fort Myers, FL | 3843 | 0.99 | 0.00 | 1.00 | 0.01 | 0.99 | 0.00 | 0.99 | 0.00 |
| Charleston-North Charleston, SC | 2149 | 0.99 | 0.01 | 0.99 | 0.01 | 0.99 | 0.00 | 0.99 | 0.01 |
| Charlotte-Concord-Gastonia, NC-SC | 1500 | 1.00 | 0.00 | 0.99 | 0.00 | 1.00 | 0.00 | 1.00 | 0.00 |
| Chattanooga, TN-GA | 1348 | 0.99 | 0.00 | 0.99 | 0.00 | 1.00 | 0.00 | 0.99 | 0.00 |
| Chicago-Naperville-Elgin, IL-IN-WI | 778 | 0.96 | 0.02 | 0.96 | 0.02 | 0.96 | 0.02 | 0.97 | 0.02 |
| Cincinnati, OH-KY-IN | 1001 | 0.98 | 0.01 | 0.98 | 0.01 | 0.98 | 0.01 | 0.99 | 0.01 |
| Cleveland-Elyria, OH | 628 | 0.93 | 0.04 | 0.93 | 0.04 | 0.93 | 0.05 | 0.92 | 0.04 |
| Colorado Springs, CO | 272 | 0.79 | 0.09 | 0.82 | 0.08 | 0.81 | 0.08 | 0.81 | 0.08 |
| Columbia, SC | 1898 | 0.99 | 0.00 | 0.99 | 0.01 | 0.99 | 0.00 | 0.99 | 0.00 |
| Columbus, OH | 791 | 0.98 | 0.01 | 0.98 | 0.01 | 0.98 | 0.01 | 0.98 | 0.01 |
| Corpus Christi, TX | 3540 | 0.99 | 0.01 | 0.99 | 0.01 | 0.98 | 0.01 | 0.99 | 0.01 |
| Dallas-Fort Worth-Arlington, TX | 2439 | 0.99 | 0.01 | 0.99 | 0.01 | 0.99 | 0.01 | 0.99 | 0.00 |
| Davenport-Moline-Rock Island, IA-IL | 904 | 0.99 | 0.01 | 0.99 | 0.01 | 0.99 | 0.01 | 0.99 | 0.00 |
| Deltona-Daytona Beach-Ormond Beach, FL | 3104 | 0.99 | 0.01 | 0.99 | 0.00 | 0.99 | 0.00 | 0.99 | 0.00 |
| Denver-Aurora-Lakewood, CO | 302 | 0.87 | 0.06 | 0.86 | 0.06 | 0.86 | 0.06 | 0.87 | 0.06 |
| Des Moines-West Des Moines, IA | 915 | 0.99 | 0.01 | 0.99 | 0.01 | 0.99 | 0.01 | 1.00 | 0.00 |
| Detroit-Warren-Dearborn, MI | 571 | 0.95 | 0.03 | 0.95 | 0.03 | 0.94 | 0.04 | 0.95 | 0.03 |
| Duluth, MN-WI | 215 | 0.72 | 0.08 | 0.76 | 0.08 | 0.74 | 0.08 | 0.74 | 0.07 |
| El Paso, TX | 1728 | 0.99 | 0.01 | 0.98 | 0.01 | 0.99 | 0.01 | 0.99 | 0.01 |
| Erie, PA | 406 | 0.84 | 0.07 | 0.88 | 0.06 | 0.85 | 0.06 | 0.88 | 0.05 |
| Eugene, OR | 126 | 0.71 | 0.07 | 0.73 | 0.06 | 0.72 | 0.07 | 0.72 | 0.06 |
| Evansville, IN-KY | 1388 | 0.99 | 0.00 | 0.98 | 0.01 | 0.99 | 0.00 | 0.99 | 0.01 |
| Flint, MI | 530 | 0.88 | 0.05 | 0.88 | 0.05 | 0.89 | 0.06 | 0.89 | 0.05 |
| Fresno, CA | 742 | 0.96 | 0.02 | 0.97 | 0.02 | 0.97 | 0.02 | 0.97 | 0.01 |
| Grand Rapids-Wyoming, MI | 524 | 0.95 | 0.03 | 0.95 | 0.03 | 0.95 | 0.03 | 0.96 | 0.02 |
| Greenville-Anderson-Mauldin, SC | 1557 | 1.00 | 0.00 | 0.99 | 0.00 | 1.00 | 0.00 | 0.99 | 0.00 |
| Hartford-West Hartford-East Hartford, CT | 526 | 0.95 | 0.03 | 0.95 | 0.03 | 0.95 | 0.03 | 0.95 | 0.03 |
| Houston-The Woodlands-Sugar Land, TX | 2903 | 0.99 | 0.00 | 1.00 | 0.01 | 0.99 | 0.00 | 1.00 | 0.00 |
| Indianapolis-Carmel-Anderson, IN | 892 | 0.99 | 0.01 | 0.99 | 0.01 | 0.99 | 0.01 | 0.99 | 0.01 |
| Jackson, MS | 2163 | 0.99 | 0.00 | 0.99 | 0.00 | 0.99 | 0.00 | 0.99 | 0.01 |
| Jacksonville, FL | 2685 | 0.99 | 0.01 | 0.99 | 0.01 | 0.99 | 0.01 | 0.99 | 0.00 |
| Kansas City, MO-KS | 1235 | 0.99 | 0.01 | 0.99 | 0.01 | 0.99 | 0.01 | 0.99 | 0.00 |
| Knoxville, TN | 1180 | 0.98 | 0.01 | 0.98 | 0.01 | 0.98 | 0.01 | 0.99 | 0.01 |
| Lakeland-Winter Haven, FL | 3413 | 0.99 | 0.00 | 1.00 | 0.00 | 0.99 | 0.00 | 0.99 | 0.00 |
| Lancaster, PA | 819 | 0.99 | 0.01 | 0.98 | 0.01 | 0.99 | 0.01 | 0.99 | 0.00 |
| Lansing-East Lansing, MI | 547 | 0.96 | 0.02 | 0.96 | 0.02 | 0.96 | 0.02 | 0.97 | 0.01 |
| Las Vegas-Henderson-Paradise, NV | 2534 | 1.00 | 0.00 | 1.00 | 0.00 | 1.00 | 0.00 | 1.00 | 0.00 |
| Lexington-Fayette, KY | 1081 | 0.99 | 0.01 | 0.99 | 0.01 | 0.99 | 0.01 | 0.99 | 0.00 |
| Little Rock-North Little Rock-Conway, AR | 1896 | 0.99 | 0.01 | 0.98 | 0.01 | 0.99 | 0.01 | 0.99 | 0.01 |
| Los Angeles-Long Beach-Anaheim, CA | 1209 | 0.81 | 0.06 | 0.82 | 0.06 | 0.82 | 0.06 | 0.81 | 0.07 |
| Louisville/Jefferson County, KY-IN | 1148 | 0.99 | 0.01 | 0.99 | 0.01 | 0.99 | 0.01 | 0.99 | 0.01 |
| Madison, WI | 585 | 0.93 | 0.03 | 0.93 | 0.03 | 0.94 | 0.02 | 0.94 | 0.02 |
| McAllen-Edinburg-Mission, TX | 4187 | 0.99 | 0.00 | 1.00 | 0.00 | 0.99 | 0.00 | 0.99 | 0.00 |
| Memphis, TN-MS-AR | 1903 | 0.99 | 0.00 | 0.99 | 0.00 | 0.99 | 0.00 | 1.00 | 0.00 |
| Miami-Fort Lauderdale-West Palm Beach, FL | 3992 | 0.99 | 0.00 | 0.99 | 0.00 | 0.99 | 0.00 | 0.99 | 0.00 |
| Milwaukee-Waukesha-West Allis, WI | 526 | 0.94 | 0.03 | 0.94 | 0.03 | 0.94 | 0.04 | 0.95 | 0.03 |
| Minneapolis-St. Paul-Bloomington, MN-WI | 586 | 0.97 | 0.01 | 0.97 | 0.02 | 0.97 | 0.02 | 0.98 | 0.01 |
| Mobile, AL | 2425 | 0.98 | 0.01 | 0.98 | 0.01 | 0.99 | 0.01 | 0.98 | 0.01 |
| Modesto, CA | 1316 | 0.99 | 0.00 | 0.98 | 0.01 | 0.99 | 0.00 | 0.99 | 0.00 |
| Montgomery, AL | 922 | 0.99 | 0.01 | 0.99 | 0.01 | 0.99 | 0.01 | 0.99 | 0.00 |
| Nashville-Davidson--Murfreesboro--Franklin, TN | 1423 | 0.99 | 0.00 | 0.99 | 0.00 | 1.00 | 0.00 | 1.00 | 0.00 |
| New Haven-Milford, CT | 569 | 0.91 | 0.04 | 0.91 | 0.04 | 0.92 | 0.04 | 0.92 | 0.04 |
| New Orleans-Metairie, LA | 2746 | 0.99 | 0.00 | 0.99 | 0.00 | 0.99 | 0.00 | 0.99 | 0.00 |
| New York-Newark-Jersey City, NY-NJ-PA | 662 | 0.92 | 0.04 | 0.92 | 0.04 | 0.93 | 0.04 | 0.94 | 0.03 |
| North Port-Sarasota-Bradenton, FL | 3484 | 0.99 | 0.00 | 1.00 | 0.00 | 0.99 | 0.00 | 0.99 | 0.00 |
| Oklahoma City, OK | 1910 | 0.99 | 0.00 | 0.99 | 0.00 | 0.99 | 0.00 | 1.00 | 0.00 |
| Omaha-Council Bluffs, NE-IA | 1018 | 0.98 | 0.01 | 0.98 | 0.01 | 0.98 | 0.01 | 0.99 | 0.01 |
| Orlando-Kissimmee-Sanford, FL | 3335 | 0.99 | 0.00 | 0.99 | 0.00 | 0.99 | 0.00 | 0.99 | 0.00 |
| Oxnard-Thousand Oaks-Ventura, CA | 560 | 0.68 | 0.08 | 0.67 | 0.08 | 0.72 | 0.09 | 0.68 | 0.08 |
| Palm Bay-Melbourne-Titusville, FL | 3416 | 0.99 | 0.00 | 0.99 | 0.00 | 0.99 | 0.01 | 0.99 | 0.00 |
| Pensacola-Ferry Pass-Brent, FL | 2462 | 0.99 | 0.00 | 0.99 | 0.00 | 0.99 | 0.00 | 0.99 | 0.00 |
| Peoria, IL | 949 | 0.95 | 0.02 | 0.94 | 0.03 | 0.95 | 0.02 | 0.96 | 0.02 |
| Philadelphia-Camden-Wilmington, PA-NJ-DE-MD | 941 | 0.98 | 0.01 | 0.98 | 0.01 | 0.98 | 0.01 | 0.98 | 0.01 |
| Phoenix-Mesa-Scottsdale, AZ | 3426 | 0.98 | 0.01 | 0.99 | 0.01 | 0.98 | 0.01 | 0.99 | 0.01 |
| Pittsburgh, PA | 588 | 0.95 | 0.02 | 0.95 | 0.02 | 0.95 | 0.02 | 0.94 | 0.02 |
| Portland-Vancouver-Hillsboro, OR-WA | 141 | 0.81 | 0.07 | 0.80 | 0.07 | 0.80 | 0.07 | 0.79 | 0.07 |
| Providence-Warwick, RI-MA | 515 | 0.88 | 0.05 | 0.90 | 0.04 | 0.89 | 0.05 | 0.92 | 0.04 |
| Richmond, VA | 1316 | 0.99 | 0.00 | 0.99 | 0.00 | 0.99 | 0.00 | 0.99 | 0.00 |
| Riverside-San Bernardino-Ontario, CA | 2773 | 0.96 | 0.02 | 0.97 | 0.02 | 0.97 | 0.02 | 0.97 | 0.01 |
| Rochester, NY | 447 | 0.87 | 0.06 | 0.87 | 0.06 | 0.87 | 0.06 | 0.89 | 0.06 |
| Rockford, IL | 713 | 0.94 | 0.03 | 0.95 | 0.03 | 0.95 | 0.03 | 0.96 | 0.02 |
| Sacramento--Roseville--Arden-Arcade, CA | 886 | 0.97 | 0.01 | 0.97 | 0.01 | 0.97 | 0.01 | 0.98 | 0.01 |
| Salinas, CA | 513 | 0.27 | 0.07 | 0.28 | 0.07 | 0.28 | 0.07 | 0.18 | 0.05 |
| San Antonio-New Braunfels, TX | 2882 | 0.99 | 0.01 | 0.99 | 0.01 | 0.99 | 0.01 | 0.99 | 0.00 |
| San Diego-Carlsbad, CA | 1311 | 0.71 | 0.08 | 0.70 | 0.09 | 0.71 | 0.09 | 0.72 | 0.08 |
| San Francisco-Oakland-Hayward, CA | 460 | 0.49 | 0.11 | 0.48 | 0.11 | 0.46 | 0.11 | 0.47 | 0.11 |
| San Jose-Sunnyvale-Santa Clara, CA | 709 | 0.76 | 0.06 | 0.76 | 0.06 | 0.78 | 0.06 | 0.78 | 0.05 |
| Santa Maria-Santa Barbara, CA | 433 | 0.32 | 0.07 | 0.33 | 0.07 | 0.33 | 0.07 | 0.24 | 0.06 |
| Santa Rosa, CA | 261 | 0.64 | 0.07 | 0.67 | 0.07 | 0.65 | 0.07 | 0.65 | 0.07 |
| Seattle-Tacoma-Bellevue, WA | 55 | 0.46 | 0.10 | 0.44 | 0.10 | 0.44 | 0.10 | 0.41 | 0.10 |
| Shreveport-Bossier City, LA | 2268 | 0.99 | 0.00 | 0.99 | 0.00 | 0.99 | 0.00 | 0.99 | 0.00 |
| Spokane-Spokane Valley, WA | 228 | 0.89 | 0.05 | 0.91 | 0.04 | 0.90 | 0.04 | 0.92 | 0.04 |
| Springfield, MA | 420 | 0.91 | 0.04 | 0.93 | 0.03 | 0.92 | 0.03 | 0.94 | 0.03 |
| St. Louis, MO-IL | 1280 | 0.99 | 0.00 | 0.99 | 0.00 | 0.99 | 0.00 | 1.00 | 0.00 |
| Stockton-Lodi, CA | 1308 | 0.97 | 0.02 | 0.97 | 0.02 | 0.97 | 0.02 | 0.98 | 0.01 |
| Syracuse, NY | 353 | 0.82 | 0.08 | 0.85 | 0.07 | 0.83 | 0.07 | 0.86 | 0.06 |
| Tampa-St. Petersburg-Clearwater, FL | 3258 | 0.99 | 0.00 | 0.99 | 0.00 | 0.99 | 0.00 | 0.99 | 0.00 |
| Toledo, OH | 787 | 0.95 | 0.03 | 0.95 | 0.02 | 0.95 | 0.03 | 0.96 | 0.02 |
| Trenton, NJ | 888 | 0.97 | 0.03 | 0.96 | 0.04 | 0.96 | 0.10 | 0.98 | 0.02 |
| Tucson, AZ | 2794 | 0.93 | 0.03 | 0.92 | 0.04 | 0.92 | 0.04 | 0.93 | 0.03 |
| Tulsa, OK | 1793 | 0.99 | 0.00 | 0.99 | 0.01 | 0.99 | 0.00 | 0.99 | 0.00 |
| Utica-Rome, NY | 247 | 0.78 | 0.07 | 0.83 | 0.06 | 0.80 | 0.07 | 0.82 | 0.06 |
| Virginia Beach-Norfolk-Newport News, VA-NC | 1458 | 1.00 | 0.00 | 1.00 | 0.00 | 1.00 | 0.00 | 1.00 | 0.00 |
| Washington-Arlington-Alexandria, DC-VA-MD-WV | 1018 | 0.99 | 0.01 | 0.99 | 0.01 | 0.99 | 0.01 | 0.99 | 0.00 |
| Wichita, KS | 1601 | 0.99 | 0.01 | 0.98 | 0.01 | 0.99 | 0.01 | 0.99 | 0.01 |
| Worcester, MA-CT | 395 | 0.87 | 0.06 | 0.90 | 0.05 | 0.88 | 0.05 | 0.91 | 0.04 |
| Youngstown-Warren-Boardman, OH-PA | 502 | 0.92 | 0.03 | 0.94 | 0.02 | 0.93 | 0.03 | 0.95 | 0.02 |

**SI References**

1. Ahn, Yoonjung, Christopher K. Uejio, Sandy Wong, Emily Powell, and Tisha Holmes. "Spatial Disparities in Air Conditioning Ownership in Florida, United States." (2022).
2. Fraser, Andrew M., Mikhail V. Chester, David Eisenman, David M. Hondula, Stephanie S. Pincetl, Paul English, and Emily Bondank. "Household accessibility to heat refuges: Residential air conditioning, public cooled space, and walkability." *Environment and Planning B: Urban Analytics and City Science* 44, no. 6 (2017): 1036-1055.
3. Gamarro, Harold, Luis Ortiz, and Jorge E. González. "Adapting to Extreme Heat: Social, Atmospheric, and Infrastructure Impacts of Air-Conditioning in Megacities—The Case of New York City." *ASME Journal of Engineering for Sustainable Buildings and Cities* 1, no. 3 (2020).
4. Guirguis, Kristen, Rupa Basu, Wael K. Al‐Delaimy, Tarik Benmarhnia, Rachel ES Clemesha, Isabel Corcos, Janin Guzman‐Morales et al. "Heat, disparities, and health outcomes in San Diego County's diverse climate zones." *GeoHealth* 2, no. 7 (2018): 212-223.
5. KEMA, Inc. 2010. 2009 California Residential Appliance Saturation Study. California Energy Commission. Publication number: CEC‐ 200‐2010‐004‐ES.
6. O’Neill, Marie S., Antonella Zanobetti, and Joel Schwartz. "Disparities by race in heat-related mortality in four US cities: the role of air conditioning prevalence." *Journal of Urban Health* 82, no. 2 (2005): 191-197.
7. Sera, Francesco, Masahiro Hashizume, Yasushi Honda, Eric Lavigne, Joel Schwartz, Antonella Zanobetti, Aurelio Tobias et al. "Air conditioning and heat-related mortality: a multi-country longitudinal study." *Epidemiology* 31, no. 6 (2020): 779-787.
8. U.S. Census Bureau. 2017 New York City Housing and Vacancy Survey Dataset. Available at <https://www.census.gov/programs-surveys/nychvs/data/datasets.html>.
